# Toward “On‐Demand” Materials Synthesis and Scientific Discovery through Intelligent Robots

**DOI:** 10.1002/advs.201901957

**Published:** 2020-02-03

**Authors:** Jiagen Li, Yuxiao Tu, Rulin Liu, Yihua Lu, Xi Zhu

**Affiliations:** ^1^ Shenzhen Institute of Artificial Intelligence and Robotics for Society (AIRS) The Chinese University of Hong Kong Shenzhen Guangdong 518172 China

**Keywords:** artificial intelligence, lab automation, on‐demand synthesis, quantum dots

## Abstract

A Materials Acceleration Operation System (MAOS) is designed, with unique language and compiler architecture. MAOS integrates with virtual reality (VR), collaborative robots, and a reinforcement learning (RL) scheme for autonomous materials synthesis, properties investigations, and self‐optimized quality assurance. After training through VR, MAOS can work independently for labor and intensively reduces the time cost. Under the RL framework, MAOS also inspires the improved nucleation theory, and feedback for the optimal strategy, which can satisfy the demand on both of the CdSe quantum dots (QDs) emission wavelength and size distribution quality. Moreover, it can work well for extensive coverages of inorganic nanomaterials. MAOS frees the experimental researchers out of the tedious labor as well as the extensive exploration of optimal reaction conditions. This work provides a walking example for the “On‐Demand” materials synthesis system, and demonstrates how artificial intelligence technology can reshape traditional materials science research in the future.

## Graphical abstract

A Materials Acceleration Operation System (MAOS) is designed for on‐demand materials discovery and synthesis. A fusion of collaborative robots, big data, virtual reality and reinforcement learning algorithms is utilized to optimize the synthesis process autonomously. Various nanomaterials with demand properties are synthesized efficiently by MAOS, indicating the vast potential of the robotic revolution in future materials research.

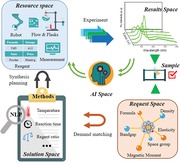

## 1 Introduction

The artificial intelligence (AI) and robotics technology are crucial for the future manufacturing; the excellent major feature is the new scheme can integrate the whole process from the virtual modeling equipment design to real‐time modulus maintenance, which provides novel and improved customer products. The experimental laboratory, as the demo stage before the industrial assembly line, requires a more complicated degree of variables and operations to reach the localized or delocalized pattern optimizations, for the functional properties. Usually, this kind of optimization processing requires a tremendous workforce, along with the side effects like safety and reproduction. There is a long history for the machine endorsed chemistry laboratory scheme, as early as Leonard Da Vinci's time.^1^ In modern time, the exploration of chemical laboratory robotics can trace backward in the 1980s.^2^ The robotics technology at that time lacks broadening intelligence and only can process limited tasks. Today, motived by the breakthrough of the AI and intelligent robots, it is right time emerging to revisit and transfer these day's advanced technology to the fundamental research areas.^3^ The preciseness of computer language and robot operation benefits a lot on solving the reproducibility problem,^4^ which has been proved both in molecular synthesis,^5^ 2D materials assembling,^6^ and nanocrystal growth.^7^ Recently, the robotics technology starts to apply in an automatic chemical laboratory vigorously widely.^8^ Big chemistry data and AI algorithms endow robots with the ability to search reaction paths and optimize experimental parameters by itself, called autonomous discovery.^9^ However, these scaling‐out methods by exploring ample parameter space are more focusing on working efficiency, less on understanding and development of scientific knowledge.^10^ The future materials discovery needs not only the robot‐assisted workforce but upgrade of methodology to make synthesis really “predictable.”^11^ The “Dial a Molecule” program in the UK and the DARPA project “Make it” in the USA are typical ongoing cases for “On‐Demand” molecule discovery.^12^ The discovery process should start from materials design, synthesis planning, automatic experiments, and characterization, to parameter self‐adjusting autonomously to achieve a closing‐loop process.^13^ It needs an intelligent brain that well coupled neuron networks, physical‐chemistry theory and scientists‐machine interactions.^14^ Since yet, most of the algorithms‐assisted synthesis system was utilized for optimizing organic molecular reaction, for example, the SNOBFIT‐assisted robotic platform in Massachu‐setts Institute of Technology (MIT),^15^ and the cloud synthesis system based on Complex Method in Cambridge.^16^ For higher demand, brains of these self‐driving laboratories are expected to extract the common concepts in experimental science, having wider compatibility, and more expansion interface.

Here, we report our intelligent robotics system Materials Acceleration Operation System (MAOS), for “On‐Demand” materials synthesis and scientific discovery. MAOS is of the E‐commerce typical like eBay or Alibaba, the user ordered the materials online, and then MAOS deliver it. A virtual lab was developed to provide a safe and practical way for man–machine interaction to train MAOS for the new experimental operations. Collaborative robot and flow chemistry system are in good cooperation for higher efficiency and degree of automation. Extensive data rate communication between lab and mobile devices works under the 4G/5G network. We include typical experiments for both of the scientific discovery and quality assurance; the synthesis of broad coverage of inorganic photoluminescent (PL) quantum dots (QDs) can sufficiently satisfy the “On‐Demand” request.

## 2 System Architecture

**Figure 1:**
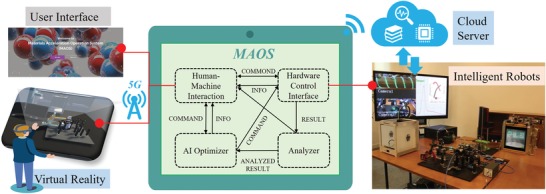
Schematic of MAOS and integrated modules, including Human–machine interaction, hardware control interface, analyzer, and AI optimizer. Users interact with robots through the website user interface (UI) and virtual reality. The cloud server provides storage, searching, and computing service for the system.

As shown in **Figure** **1**, the human–machine interaction module provides an input window for users to submit demands of various properties; the user also can control and monitor the system online. Currently, we support a user's user interface (UI) of web and virtual reality (VR) (the left part in Figure 1) to interact with MAOS. The Hardware control interface (right part in Figure 1) managed all hardware modules and provided a set of independent hardware instructions for AI optimizer and the system manager. The Analyze module within MAOS first receives the raw data from the characterization modules, automatic analyze, and optimize the experiment parameters to meet the demand on request under reinforcement learning (RL) scheme. After the precision criterion converged, it sends the feedback to the optimizer and visualizes the results to the user (The workflow of MAOS is in Section S1, Supporting Information). Cloud sever was utilized for data storage, searching, and high‐performance computing if necessary. Due to the high amount data flow including data and video, the WIFI and 4G/5G communication between local lab and mobile devices are essential; we also have tested the 5G signal for the time delay in the remote control of MAOS through mobile phone.

### 2.1 Workflow Throughout Various Space

**Figure 2:**
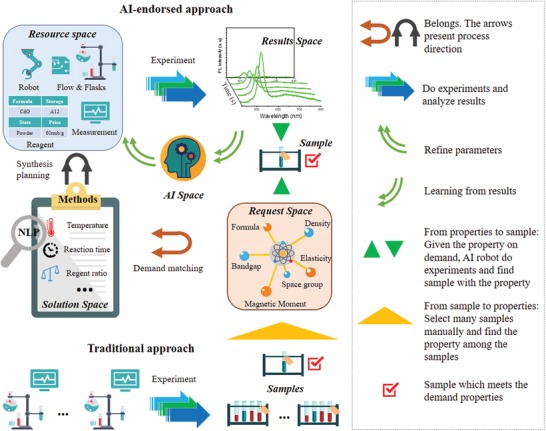
Comparison on the workflow of AI‐endorsed “On‐Demand” materials discovery and traditional approach, legends for concept explanation is on the right side. For the AI‐endorsed approach, the procedure starts by identifying target materials according to user demand properties in request space. Then, demand matching between request space and solution space was made refer to the synthesis methods database. NLP benefits a lot here in extracting keywords from journal articles and arrange them. Synthesis planning will be made by utilizing available modules in the resource space. Both request and resource space should belong to the solution space to verify the workflow keep going. Thus the virtual screening process ends, and experiments take place. In situ experimental feedback is stored in results space and flow to AI space as a learning dataset. The refined parameters will be updated for the next experiment. The closed‐loop workflow constituted by resource space, result space, and AI space will take time to approach the optimal parameters and obtain the target sample. The whole process is distinguished from the traditional way for materials research, which starts from scale‐up parallel synthesis and screens out very limited samples to meet the demand.

The workflow of “On‐Demand” materials synthesis could be described as data and matter flow throughout various Spaces, shown in **Figure** **2**. The yellow triangle arrow indicates the traditional paradigm of materials synthesis, which works from samples to properties, it first synthesis amounts of samples, and picked up only the excellent ones after property check, the off‐target ones are usually discarded. The green triangle arrow indicates the “On‐Demand” paradigm, based on the AI and Robots technology; it works from properties to samples, the off‐target ones seldom came into being, which saves significant resources in environments and economics. The infrastructure is constructed by the individual database including materials properties database, reagent database, methods database, machine database, and results database (Details in Section S2, Supporting Information). MAOS stores long‐term data through SQLAlchemy,^17^ which supports many types of database management system, such as MySQL, Postgres, Oracle, and SQLite. MySQL on the cloud server is used to store the data. In the “On‐Demand” materials synthesis idea, MAOS automatically finds the target materials through the database to satisfy user's requirement on the materials property, and on the other side, MAOS screens out available ones according to the synthesis ability of local laboratory. The online database, such as Open Quantum Materials Database^18^ is linked for the target materials with property searching. This virtual screening process throughout the Request Space, Solution Space, and Resource Space to generate synthesis planning, as shown in the left part of Figure 2. The Solution Space is essential for the automatically experimental recipe generating. It includes the database of reactant types, concentration, volume/weight, and reaction conditions. Natural language processing (NLP)^19^ technology is implemented here for the construction of the method space based on the previous research by Olivetti et al.^20^ Next, MAOS will check whether the laboratory has adequate synthesis modules, reagent storage, and characterization equipment in Resource Space for the required solution. For example, chemical vapor deposition, molecular beam epitaxy are not feasible for the current version of MAOS, hydrothermal method, sol–gel method, and most of the solution‐based process can be well satisfied. A pricing model drew on design principles of green chemistry announced by American Chemical Society (ACS)^21^ was applied for optimal planning (Section S3, supporting information). The index indicates toxics, atom economy, safety, and renewability in the model. After the virtual screening, MAOS will load the history experiment results from Result Space. If no such database exists, the robots will start the first experiment with initial parameters generated in AI Space. The in situ characterized experimental data are stored in Result Space (right top in Figure 2), and then sent to AI Space for optimization. An optimizer in AI Space will decide parameters adjustment for the next loop and finally approach the demand target (the detail of the optimization is shown in the Experimental Section; and Section S4, Supporting Information). Movie S1 in the Supporting Information shows the whole workflow of “On‐Demand” material synthesis.

### 2.2 Human–Machine Interaction

**Figure 3:**
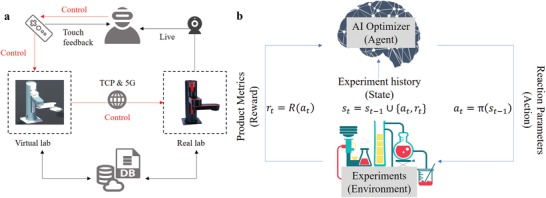
Scheme of VR‐based remote control of robots and RF learning model in materials synthesis. a) Remote users with VR glasses and gamepad operate the robot in a virtual lab. The robot in the real lab was guided refer to position information of virtual robot. b) Basic concepts in RF learning model including agent, action, environment, and reward are well coupled in materials synthesis space and played a role. *s_t_* is all the information about the reaction space. *a_t_* is the experimental condition determined by policy function π. *r_t_* is the reward of reaction R under *a_t_*.

MAOS provides two efficient ways for users to communicate with the system, through UI‐Web (UI: user interface) or UI‐VR devices. The UI‐Web built on cloud server has two channels for the users and sellers (materials supplier or administrator), respectively. The interface for users is similar to an online shopping website like eBay or Alibaba; users can search target materials according to the request on materials properties, like the bandgap, then order after the price checking (As shown in the starting part of Movie S1, Supporting Information). MAOS will first generate the operation recipe once received the order, if the synthesis plan of the target material is in the record, MAOS then started the synthesis automatically; if the experiment is new to MAOS, the administrator can remotely operate the lab through VR and train MAOS for the whole experimental operation details. The UI–VR interface is a virtual lab and isomorphic reflection of the real lab. As shown in **Figure** **3**a, the administrator can get sight and control a virtual robot in the lab through VR equipment. The virtual robot communicates with reality through 5G network or transmission control protocol (TCP) based protocol (Section S5, Supporting information). The virtual robot could translate user command to controlling command for the real robot in real‐time. For some hard experimental operations jobs, the commands could be executed in a virtual experiment first and then executed in a real environment. Cameras recording the real lab information enable the administrator to get a live stream of the virtual and real lab at the same time in VR glass for improved training. In UI–VR, the robot arm is forbidden to collide with other equipment. When administrators training robot arm to do jobs (such as grasping beakers) in VR, each job and all related commands would be recorded and facilitated via database. With the post‐trained data, the robot can well repeat the same task in the record. The VR and collaborative robot constitute a pivotal module to replace the tedious labor works. The detail of the robot arm structure and parameters structures with VR are shown in Section S6, Supporting Information.

### 2.3 Analyzer and AI Optimizer

The synthesis process generates high‐throughput feedback, including video frame flows and in situ spectrum by characterization modules, which occupies hundreds of million bits per second data rate. Before sent to the optimizer, these feedback data need preprocessing into certain forms by extracting demanded information to verify the learning efficiency of the optimizer. An efficient automatic analyzer for data processing is necessary for such a self‐driving system. Analyzing models for Computer Vision (CV) and spectrometer were developed for real‐time data processing. The CV analyzer based on OpenCV^22^ library was applied to extract red, green and blue (RGB) information from video streams captured by the in‐built camera, and then mapped it into the average particle size 〈*D* ^RGB^〉 or 〈*D*〉 From a well‐benchmarked machine learning scheme. Details of the analyzing algorithms were described in our previous work.^7^ The spectrum analyzer is used to extract the demand optical information from spectrum files generated by spectrometer software. Center wavelength and intensity of peaks was first identification. Then multipeak Gaussian fitting is applied based on SciPy.^23^ The emission wavelength and FWHM (Full width at half maximum) of the product are calculated from a Gaussian curve with maximum peak value.

As shown in Figure 3b, an AI optimizer acts as a decision maker to adjust reaction parameters through the whole procedure of synthesis. A trial and error process guided by searching algorithms endorse the system ability of autonomous discovery. A reaction could be viewed as a system taking multiple inputs (parameters generated by all hardware modules) and return multiple outputs (product property metrics, such as emission wavelength and FWHM). Optimizer needs to search the experimental parameter space and find the state with demanded properties, with the minimum number of steps. Here we apply the RL scheme, which focuses on how to maximize the “reward” from “environment” through “action,”^24^ which proved influential in optimizing chemical reaction.^25^ For materials synthesis, the reaction is the “environment,” an algorithm decides the experiment parameters to try “actions” in order to find the desired product (reward). For MAOS, the RL process was guided by SNOBFIT algorithms,^26^ which has been applied inorganic^27^ and inorganic^28^ synthesis optimization. Detailed algorithms implementations, including the language, parameter spaces, and operators are in the Experimental Section.

### 2.4 Hardware Modules and Control Interface

**Figure 4:**
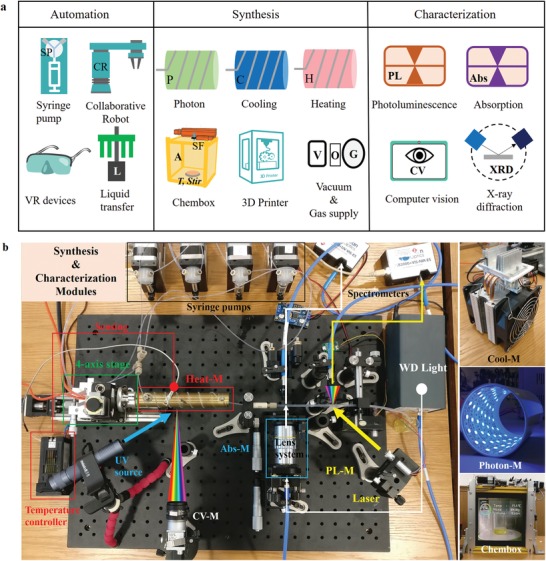
Available reconfigurable hardware modules in MAOS. a) Syringe pumps, collaborative robot, VR system, and a liquid handler were coupled to realize multiple types of automation. The functional modules, including vacuum or N_2_ atmosphere, stir, temperature gradient, and light radiation. A 3D‐printer is efficient for printing specifically designed components. The PL, Abs, and CV modules collect the in‐line photoluminescence, optical absorption spectrum, and computer vision data, respectively. XRD (X‐ray diffraction) module work for phase identification of synthesized crystalline material. b) Photograph of assembled modules for typical materials synthesis and characterization. Various experimental conditions and operations can be realized by assembling the relevant modules.

As shown in **Figure** **4**a, in MAOS, hardware modules can be divided into three parts by function: for automation, synthesis, and characterization. A challenge of traditional flow chemistry process is that the reagents are presettled and connected with pumps, tedious human labors are inevitable for the reactants preparation work. In MAOS, a collaborative robot was introduced in to handle these repetitive works. At the same time, a virtual lab is constructed to train the robot or control it in real time by remote administrators. The robot arm can handle the beakers to a liquid transfer module, which is a designed liquid handler made up with stepping motor and 3D‐printed components. Thus the connection between solution container and flow system links automatically. The heating (Heat‐M, marked in red), cooling (Cool‐M, right top in Figure 4b), and photon module (Photon‐M, right middle in Figure 4b), provides variant experimental conditions for the investigation of external field perturbation on the synthesis. The cooling model controlled by a proportional integral derivative (PID) device and a K‐type sensor, provides the temperature ranges from −20 to 300 °C; The photon module includes a 30W UV irradiation (365 nm). The Chembox combined the electronic weighting module, temperature measurement, and an intelligent interface, which displays the inside chamber view and chemical information, including solution volume, weight, reaction temperature, and stirring speed. A vacuum‐nitrogen supply system also couples with Chembox, which covered the function of traditional double row tube in chemical synthesis. A 3D printer is used to manufacture small batches of customized structures, such as sample holders and low‐temperature reactors. For characterization, photoluminescence (PL‐M, marked in yellow), absorption (Abs‐M, marked in blue), and computer vision (CV‐M, marked in white) are utilized for in situ monitoring the optical properties of synthesized materials. Details of hardware modules are in Section S6, Supporting Information.

All robotic automation and characterization modules are controlled by a hardware interface in MAOS, which includes both high‐level and low‐level instructions based on JSON‐RPC2.0 protocol. The high‐level instruction is a formula made up of instruction symbols and operators. Each symbol indicates the corresponding hardware module, and each module has its specific parameter vector, which represents all adjustable parameters this module can provide. Taking an example for the Heating module, the instruction symbol is H, and the parameter vector is $\left[Th,\;\dot{T}h,t^{H}\right]$. Here, *Th* is the stable temperature controlled by H, $\dot{T}h$ is the temperature gradient (heating rate), *t^H^* is the working time of H. The symbol array collected by operators can describe the arrangement of modules and reagent in both time and space domain. Five kinds of operators are utilized: “+”,“×”,“⋅”,“|”, and “∪” . By combining different module symbols and the operators, a language is developed for the communication between MAOS and the real experiments. Details of all instruction symbols, parameter vector (Table 2) and operators (Table 3) can be found in the Experimental Section. With the help of the language, all experimental operations can be interpreted by a formula, and then been converted into the machine code, which is defined as process of “compile” (details are shown in Section S6 and Figure S2, Supporting Information). All parameters in an experiment will be stored in vectors to construct the parameter space, which is called “parameterization.” These vectors are input data for both hardware control interface and AI optimizer.

## 3 Results and Discussion

### 3.1 “On‐Demand” Synthesis of Colliding QDs with Target Emission Wavelength and FWHM

Next, we introduce the whole procedure of the “On‐Demand” synthesis scheme in MAOS. One of the most urgent desires for this kind of technology lies in the optoelectronic QDs, which are promising materials for application fields in the display, lighting, and sensing devices.^29^ These applications often demand precise nanoscale‐engineering on component ratio, crystal size, and size distributions to verify target emission wavelength and FWHM. These two parameters well correlate with the QD size^30^ and the size focusing ,^31^, ^32^, ^33^ thus the “property On‐Demand” issue in the optoelectronic QDs system can be equally interpreted into the “size and distribution On‐Demand” issue. However, it is still a challenging issue for controlling both of the two quantities in the same time because, theoretically, the diffusion‐limiting growing mechanism usually comes along with the Ostwald ripening, which always broadening the size distribution^32^ and lowering the QDs quality.

**Figure 5:**
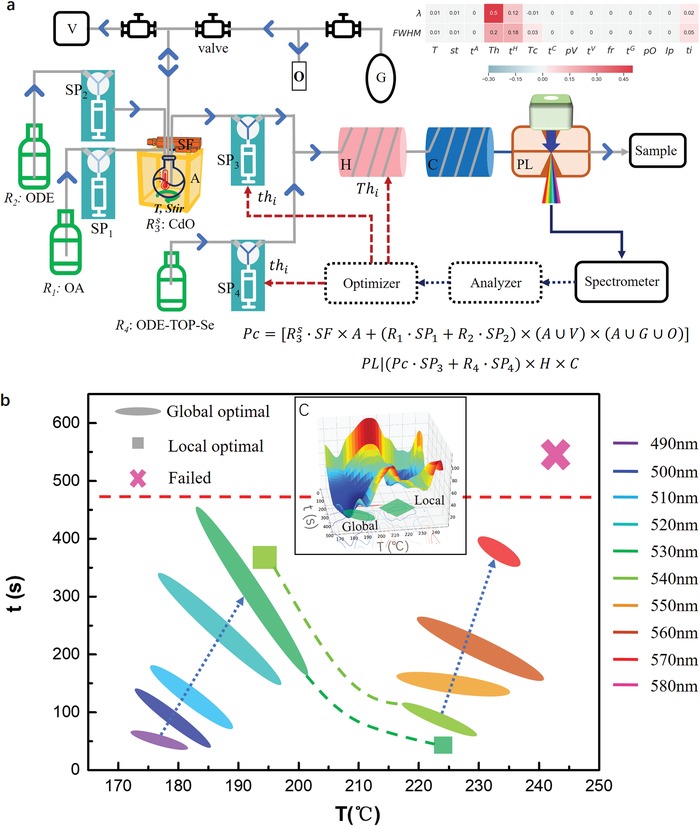
Synthesis schematic and optimization process in the “On‐Demand” synthesis of CdSe QDs with target emission wavelength and FWHM. a) Assembled modules for multistep synthesis. Two syringe pumps and the heating module was utilized to optimize the reaction condition under control of optimizer (blue arrow indicates the flow direction, solid lines, and dashed lines represent matter and information flow). The top right inset image shows the correlation of all parameters with results. b) “On‐Demand” synthesized CdSe QDs with different λ_*t*_ mapping on the 2D parameter $\left[Th_{1},t_{1}^{H}\right]$ plate. Color of icons indicate λ_*t*_ (see legends on the right). Ellipses on this plate indicate the parameter region which achieved λ_*t*_ ± 0.5 nm and FWHM_*t*_ < 35 nm, also described as global optimal solution $\left[Th_{g},t_{g}^{H}\right]$. The blue arrows indicate the trend of $\left[Th_{g},t_{g}^{H}\right]$ along with the increase of λ_*t*_. Local optimal solution $\left[Th_{l},t_{l}^{H}\right]$ (marked as cubic) were discovered when approaching 530 and 540 nm demand. Optimization failed when λ_*t*_ = 580 nm. The red dash line is a boundary that no solution exists for FWHM_*t*_ < 35 nm. c) The 3D viewers of the global and local optimal position for target λ_*t*_ = 540 nm.

Here, in this case, we apply the MAOS to further improve the QDs quality by satisfying the demand. The synthesis planning made by MAOS suggested a hot injection stratagem for CdSe nanocrystal utilizing multiple synthesis modules, as shown in **Figure** **5**a. The available hardware modules endorsed a fully automatic synthesis workflow. Vacuum pump and N2 flow controlled by a solenoid valve provided an adjustable gas environment for ODE‐Cd precursor preparation. Uniform stirring and temperature control are integrated into this flask (in yellow) based system in Chembox, where the ODE‐Cd and TOP‐Se precursors were well prepared, then the syringe pumps injected the liquid through the multistep reaction modules, the flowing direction is indicated by the arrow in Figure 5a (more experimental details are in Section S7, Supporting Information). The MAOS language for this experiment includes two steps

(1) $$\text{Pc}\;=\;\left[R_{3}^{s}\cdot \text{SF}\times A+\left(R_{1}\cdot {\text{SP}}_{1}+R_{2}\cdot {\text{SP}}_{2}\right)\times \left(A\cup V\right)\times \left(A\cup G\cup O\right)\right]$$

(2) $$\text{PL}|\left(\text{Pc}\cdot {\text{SP}}_{3}+R_{4}\cdot {\text{SP}}_{4}\right)\;\times H\times C$$

Equations (1) and (2) interpret the Robots arm action in reactants preparation and PL measurements, respectively. Before optimization, we applied a parameter correlation test on all adjustable parameters in this experiment; as shown at the right top in Figure 5a. In this test, a default parameter vector was chosen as the baseline; the individual change of each parameter with 20% variation was applied. We find only two parameters $\left[Th_{i},t_{i}^{H}\right]$ correlate more than 10% within the characterization results (λ and FWHM), which indicates only the heating module (H) matters here. As shown in the blocks in Figure 5a, the heat module (block H in Figure 5a) controlled the reaction temperature, which is parameterized as *Th*, with *t^H^* is the working time of H; and the reaction time (t) was well affected by the sum of injection rate τ of the pump,^33^ which is parameterized as action *a_i_*, can be represented as action $a_{i}=[Th_{i},t_{i}^{H}]$, which are independent parameters for the optimizer. Once the experiment started, a random $\left[Th_{0},t_{0}^{H}\right]$ parameters are initiated for step 0 as in the subscript, MAOS first collected the emission spectrum (block PL in Figure 5a) of the continues synthesized samples data from the spectrometer, checked the validation of the PL signal, then sent the spectrum data to the Analyzer block for information extraction, in this stage, the user demand properties (PL center wavelength (λ_*t*_) and target FWHM (FWHM_*t*_) of synthesized materials were extracted and compared with the experimental one in the current stage; if the criterion converged, the experiment steps, or else, MAOS activated the optimizer block, updating the $\left[Th_{0},t_{0}^{H}\right]$ to $\left[Th_{1},t_{1}^{H}\right]$, iterated to the criterial convergence.

Statistic results of all optimal $\left[Th_{i},t_{i}^{H}\right]$ for λ_*t*_ ranges from 490 to 530 nm, are shown in Figure 5b, and the data are summarized in **Table** **1**. We can see that multiple parameter sets $\left[Th_{i},t_{i}^{H}\right]$ can satisfy the user's demand in each ellipse plane in the 2D plot. Each ellipse was established by one to three experimental results within the region (original data are in Figure S13, Supporting Information). An example of the visualization of the optimization process for a typical experiment (λ_*t*_ = 530 nm, FWHM < 35 nm) is inset as Figure 5c. RL algorithms guided the parameter optimization process. Here the RL defined action is $a_{i}=[Th_{i},t_{i}^{H}]$ and the reward is *r_i_* = [Δλ_*i*_,ΔFWHM_*i*_] = [λ_*t*_ − λ_*i*_,FWHM_*t*_ − FWHM_*i*_] . We define the loss function as L=abs(Δλ)+20*ReLu(−ΔFWHM), representing the difference from the target. L<0.5 was set as converge condition. For RL optimization, the numerical range of *Th_i_* and *t^H^* are within(170 °C, 245 °C) and (20s, 600s) respectively, as illustrated as the X and Y axis in Figure 5b. The z‐axis illustrates the trials to reach the convergence. Since every step MAOS will redo the whole experiment again, it takes about 4 h to close the optimization. We can see that a locally optimal solution $\left[Th_{l},t_{l}^{H}\right]$ (green square) was first discovered by MAOS. Then, the global searching function overcame the barrier and discovered a better‐converged parameter set $\left[Th_{g},t_{g}^{H}\right]$ in a higher temperature region (green ellipse), which is different from cases that λ_*t*_ < 530 nm. As shown in Figure 5b, the trend of $\left[Th_{g},t_{g}^{H}\right]$ along with the increase of λ_*t*_ is to increase both *Th_g_* and $t_{g}^{H}$. Overall, the *Th_g_* changes more than the $t_{g}^{H}$ during the optimization. However, when $t_{i}^{H}$ approaches the red dash line around *t* = 470s, the emerging of Ostwald Ripening strongly limited the further optimization of FWHM, which makes the long reaction time (large t) a forbidden choice for FWHM optimization, MAOS will turn the temperature dimension for the numerical solution. This physics picture caused the local optimal state as shown in the green square for the case λ_*t*_ = 530 nm. For larger λ_*t*_, the search of $\left[Th_{i},t_{i}^{H}\right]$ mainly focus in the high‐temperature region. Thus, for λ_*t*_ = 540 nm, there is no optimization solution bellow 200 °C; the previous local optimal solution in the high‐temperature region became the global optimal one. In this jumping process, MAOS tune the value of *th_i_* by reducing the reaction time *t* from 370s to near 75s, resulting in a 5 nm drop of FWHM, and the temperature increase from 195 to 225 °C, as indicated by the λ_*t*_ = 540 nm data in Figure 5b.With further increasing of λ_*t*_, the trend of $\left[Th_{g},t_{g}^{H}\right]$ approaches the red dash line; however, since the high temperature can destroy the chemical bonding in QDs, the temperature range is limited, which does not allow another jumping to the solution region. Thus there is no solution for λ_*t*_ = 580 nm and above with the FWHM < 35 nm constrain.

**Table 1:** A summary of partial achieved results under reinforcement learning with different target wavelength, the data are ordered by sequence in MAOS

Target λ [nm]	Optimal λ [nm]	Error [nm]	FWHM [nm]	Trials	Temperature [°C]	Time [s]
490	489.8	0.2	33.8	56	175	56
500	499.6	0.4	34.6	25	180	98
510	509.6	0.4	33.2	16	180	156
520	519.8	0.2	31.4	8	190	208
530	529.6	0.4	34.0	2	195	304
540	539.6	0.4	34.6	2	225	76
550	549.8	0.2	34.6	1	225	152
560	560.4	0.4	34.6	4	230	210
570	569..8	0.2	34.8	15	235	356
580	580.4	0.4	39.8	100 (Max)	245	520

During the whole optimization, MAOS can record the parameter details, for the following task, for example, 535 nm target with 35 nm FWHM, as shown in Table 1, it only takes 25 steps for reaching the convergence. Moreover, based on the post‐training steps from the previous task, it only takes two trials for the fifth task as 520 m and 35.0 nm target, which provides an excellent demo for the following “On‐Demand” tasks.

Even though the above solution is of a pure numerical style, it still reveals insightful information inside the optimization details. We now understand that it is challenging to synthesize the large size QDs with narrow size distribution due to the limitation of the nucleation theory as discussed above,^31^, ^32^ a more comprehensive additional 〈*R*〉 dependent subsection policy is essential for the solution of this issue to satisfy the demand on QDs of large radius.

**Figure 6:**
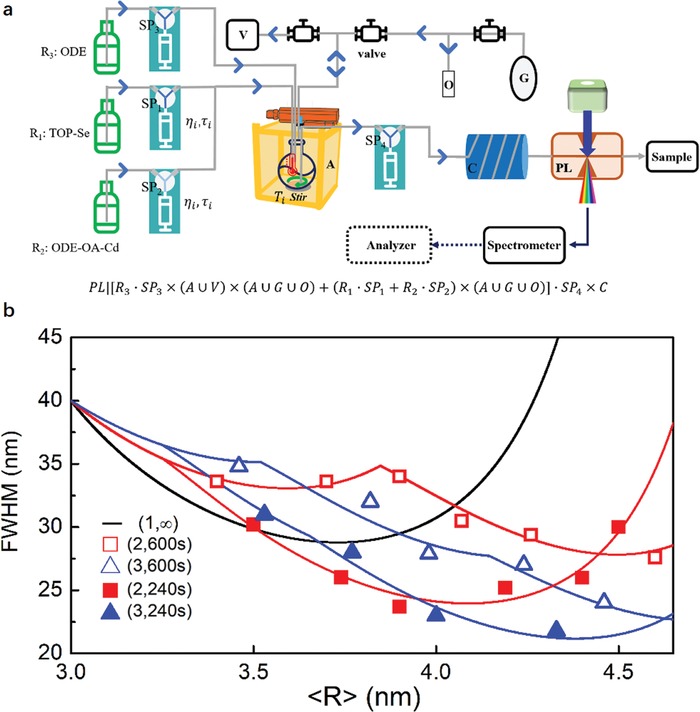
Scheme of synthesis strategy provided by assembled modules and sketch for the multi‐injection mechanism. a) CdSe QDs were synthesis in Chembox and continuously extracted through Cooling and PL modules for measurement. b) FWHM~〈R〉 dependence plot of the nanoparticles produced, with (η, τ) groups (2, 240*s*), (2, 600*s*), (3, 240*s*), and (3, 600*s*). The black line indicates the typical Ostwald Ripening, the red and blue line fit the data of η = 2, 3 cases, respectively.

Previous work reported that the additional injection during the growth could significantly improve the size distribution.^7^, ^33^ Here we apply MAOS by variant injection time interval τ and the times of the injection operation η, and we keep the total injection amount η*c_η_ constant for different η value . The experimental setup in MAOS is shown in **Figure** **6**a, the MAOS language here is

(3) $$\text{PL}|\left[\begin{array}{l} R_{3}\cdot {\text{SP}}_{3}\times \left(A\cup V\right)\times \left(A\cup G\cup O\right)+R_{1}\cdot {\text{SP}}_{1}\\ \;\;+R_{2}\cdot {\text{SP}}_{2}\times \left(A\cup G\cup O\right) \end{array}\right]\cdot {\text{SP}}_{4}\times C$$

The difference from Figure 5a is the reaction location now is Chembox, which confines all the reactions space. Since we investigate how the interval injection can affect the QDs quality through diffusion in‐between the total QDs, a uniform reaction space is essential. Figure 6b shows the experimental data for the average radius (〈*R*〉) and FWHM with η and τ dependence which looks like chaos at a first glimpse. For the two cases (2, 600s) and (2, 240s), as shown by the empty and solid cube in Figure 6b, we can see there is an apparent radius dependent of FWHM character. The (2, 240s) has favorite 〈*R*〉 when 〈*R*〉 < 4.4 nm, for 〈*R*〉 > 4.4 nm, the FWHM from (2, 600s) strategy results in more narrow FWHM and QDs quality. This experimental phenomenon indicates that the injection interval τ can be an efficient operational parameter in the MAOS to optimize the size‐dependent FWHM. We reset the τ value extending to the (3, 240s) and (3, 600s) cases, as shown in the empty and solid triangle in Figure 6b, we observed the same picture as the (2, 240s) and (2, 600s) cases, the critical radius enlarged to 4.7 nm. Since the transition from σ to FWHM can be linearly transferred as $\text{FWHM}\;=\;2\sqrt{2}(2\log2)\sigma$, the analytic model of the (η, τ) correlated 〈R〉~FWHM dependence can be derived as

(4) $$\;\frac{{\text{FWHM}}_{t}}{{\text{FWHM}}_{0}}={\left(\frac{{\left\langle R\right\rangle }_{t}}{{\left\langle R\right\rangle }_{0}}\right)}^{\frac{1}{\xi_{t}}-1}\;={\left(\frac{{\left\langle R\right\rangle }_{t}}{{\left\langle R\right\rangle }_{0}}\right)}^{\frac{1}{\xi_{0}\text{exp}\left(-\frac{t}{\mu }\right)\left(1-\text{exp}\left(\frac{\eta \tau }{\mu }\right)\right){\left(1-\text{exp}\left(\frac{\tau }{\mu }\right)\right)}^{-1}}-1}$$

Here ξ_0_ is a constant, 〈*R*〉_*t*_ and 〈*R*〉_0_ is the average radius at time *t* and initiate state, µ is the distribution length defined in the reaction domain of each injection, the detail of the derivation is in Section S7 (Supporting Information). Figure 6b shows the main numerical results for an operational (τ, η) dependent 〈R〉~FWHM plots, as mentioned in the previous part. The chaos‐like discrete experimental data can be well fitted by Equation (4), indicating the robust solution for the 〈*R*〉 dependent FWHM optimization. Due to the well‐integrated modules in MAOS, the plentiful parameter spaces cooperated hardware can afford long‐time consistent work with high precision and reproduction, provides more possibilities for discovering new physical and chemical properties than the traditional experimental operation. With the sufficient high‐quality experimental data, MAOS can work as an excellent platform for inspiring in‐depth understanding from theoretic level, as shown in Equation (4). Like in this case, the “On‐Demand” functions can be further benefited from the feedback of theoretical endorsement, MAOS also can work for the optimization of QDs quality with large size through the time‐dependent interval injection strategy. Section S8 (Supporting Information) summarizes several optoelectronic, photothermic inorganic nanomaterials with which can be synthesized in MAOS, which provides a uniform paradigm toward the “On‐Demand” synthesis.

## 4 Conclusion

To conclude, MAOS was systematically architectured with language, compiler, and operation modules. For the general experiments, MAOS can separate the human‐being from the lab location through the VR‐robot interaction through the 4G/5G network, and for the post‐trained experiments, MAOS can finish the experimental task by fully automatic procedures with AI assured quality control, reducing the time cost to 1/20. We also highlighted the robustness of MAOS for optimizing both the emission wavelength and FWHM quantities in CdSe QDs. A theoretical description for the wavelength dependent optimal strategy can be derived and fed back to MAOS, which makes the “On‐Demand” synthesis feasible for the optoelectronic QDs more than CdSe (see Section S8, Supporting Information). MAOS provides a transparent paradigm for future materials science, which is of more safety, efficiency, and intelligence.

## 5 Experimental Section

##### The AI Space

The parameters for the RL modules in AI space in this work is of the form like $\left(S,A,\{P_{as}\},R\right)$. Here S denotes the set of state *s*, which is the set of all reaction parameter and an experimental result that has been experimented. State at timestamp *i* is denoted by *s_i_* = {*a*_0_,*r*_0_,…, *a_i_*,*r_i_*}; A denotes the set of action *a_i_*. In the context of reaction optimization. A is the combinations of reaction parameters, such as reaction time and temperature. Action at timestamp *i* is denoted by $a_{i}=[a_{i}^{1},\ldots ,a_{i}^{j},\ldots ,a_{i}^{n}]\;;$ $\left\{P_{as}\right\}$ denotes the state transition probability, here *p_as_* decides what action *a* to make under the experimental condition s. This action decision policy is denoted by π; R denotes the reward function under state S and action A. In the environment of reaction optimization, the reward at timestamp *i* is denoted by $r_{i}=[r_{i}^{1},\ldots ,r_{i}^{j},\ldots ,r_{i}^{n}]$, the reward function denotes the mapping *r_i_* = *R*(*a_i_*). At a specific experimental condition *a_i_* is mapped to get the difference between the experimental and the target then get the reward *r_i_*.

For a timestamp *i* with the previous state *s*_*i* − 1_, the policy function decides the action to make by *a_i_* = π(*s*_*i* − 1_). Then, continue the experiment with *a_i_*, and get the reward *r_i_* = *R*(*a_i_*). For the MAOS, architecture, the state could be updated by *s_i_* = *s*_*i* − 1_ ∪ {*a_i_*, *r_i_*}. Policy function π performs local optimizations around the best conditions while searching for unexplored regions to ensure global optimality. For local optimization, the action is decided by searching from the best condition with a full quadratic model. For example, an action could be decided by approximately minimizing the local quadratic model. $q\;(a)=r_{\text{best}}\;+g^{T}(a-a_{\text{best}})+\frac{1}{2}{\left(a-a_{\text{best}}\right)}^{T}G(a-a_{\text{best}})$, where *g* and *G* are the gradients and the symmetric matrix estimated, respectively. 26

For the global optimization, the action is decided by searching simultaneously in several promising subregions. For example, the reaction space is partitioned into sub‐boxes each contains a history state.^26^ The actions are chosen by

$a=\left\{ \begin{array}{l} \frac{1}{2}(\underline{a_{i}}+a_{i}),\;\text{if}\;a_{i}-\underline{a_{i}}>\bar{a_{i}}-a_{i}\\ \frac{1}{2}(\bar{a_{i}}+a_{i}),\;\text{otherwise} \end{array}\right.$, where $\left[\underline{a_{i}},\bar{a_{i}}\right]$ is one of the largest sub‐boxes of the current partition with history action *a_i_* inside. The actions selected will finally be rounded into the nearest integral multiple of the resolution vector Δ*a*, which is decided by the physics condition of reaction parameters (here $\Delta \;a\;=(\Delta Th_{i},\Delta t_{i}^{H})\;=\;(5{\;}^{{}^{\circ}}\text{C},3s)$, *Th_i_* and $t_{i}^{H}$ are the heating temperature and heating time, respectively). A specific reaction parameter Δ*a^j^* large enough can be made, then the local optimization would have little influence on this dimension because of the round procedure. For global search mentioned above, new actions would be selected in the most massive sub‐boxes.

##### Specification of Instruction Symbols in MAOS

**Table** **2** symbolizes and parametrizes all the modules in MAOS, with time dependence. ***θ*** is defined as the vector that concludes all adjustable parameters as

(5) $$\begin{array}{c} \theta =[T,st,t^{A},W,F,t^{P},Th,\dot{T}h,t^{H},Tc,\dot{T}c,t^{C},pV,t^{V},fr,t^{G},pO,t^{O},\\ \text{exit},Ip,Ia,r,fps,et,vi,ti,\eta ,\tau ,wi,tf,P_{i},P_{f},tr,\text{state}] \end{array}$$

**Table 2:** Specification of instruction symbols and parameter vectors in MAOS

Symbol	Module	Parameters	Parameter vector
*A*	Chembox	Temperature, stirring speed, time	[*T*, *st*, *t^A^*]
P	Photonic module	Power intensity, wavelength, time	[*W*, *F*, *t^P^*]
H	Heating module	Temperature, temperature gradient, time	$\left[Th,\;\dot{T}h,t^{H}\right]$
C	Cooling module	Temperature, temperature gradient, time	$\left[Tc,\;\dot{T}c,t^{C}\right]$
V	Vacuum pump	Pressure, time	[*pV*, *t^V^*]
G	Gas supplier	Gas flow rate, time	[*fr*, *t^G^*]
O	Gas treatment	Pressure, time	[*pO*, *t^O^*]
PL	PL measurement	Excitation source, integration time	[*exit*, *Ip*]
Abs	Absorption measurement	Integration time	[*Ia*]
CV	Computer vision	Resolution, fps, exposure time	[*r*, *fps*, *et*]
SP	Syringe pump	Injection volume, time, injection rate, times of injection	[*vi*, *ti*, η, τ]
SF	Solid feeding module	Feeding weight, feeding time	[*wi*, *tf*]
CR	Collaborative robot	Initial point, final point, time	[P_*i*_, P_*f*_,tr]
L	Liquid transfer	State (up or down)	[state]

The ***θ*** vector delivers orders to all the modules in MAOS for the given project, and it also guarantees the repeatability for the typical task. The materials, like reagents, are also parametrized to make the whole system digitalized. *R_i_* is defined the liquid reagent, as *R_i_* = [*i*, *v_i_*]. $R_{i}^{s}$ the solid reagent, as $R_{i}^{s}=\;[i,\;w_{i}]$. Here *v* is the volume of liquid reagent, *w* is the weight of solid reagent, *i* is the serial number of the reagent in database. MAOS conduct the experimental operation by combining the parameter spaces of modules and the chemical reagent, and an operator is essential to connect the two parameters space. As shown in **Table** **3**, there are four well‐defined operator symbols with the representation and examples, and each operator correlates the symbolic calculations within the module and chemical parameters. For example, *R*_1_ · SP_1_ can be interpreted as MAOS transfer the liquid chemical *R*_1_ by the first Syringe pump, as indexed SP_1_ from Table 2.

**Table 3:** Meaning and usage of operators

Operator symbol	Representation	Usage example
·	Reagent transfer	*R*_1_ · *SP*_1_ (transfer *R*_1_ with *SP*_1_)
+	Fusion of reagent	*R*_1_ + *R*_2_ (Adding *R*_2_ into *R*_1_)
×	React with environment	*R*_1_ × *A* (*R*_1_ react in Chembox)
|	Characterization	*PL*|*R*_1_ × *A* (Take PL measurement of *R*_1_ in Chembox)
∪	Condition combination	*R*_1_ × (*A* ∪ *V*) (*R*_1_ react in Chembox with vacuum environment)

## Conflict of Interest

The authors declare no conflict of interest.

## Supporting information

Supporting Information

Supplemental Movie 1
