# Supplementary material for: Toward “On‐Demand” Materials Synthesis and Scientific Discovery through Intelligent Robots
Source: Adv Sci (Weinh). 2020 Feb 3;7(7):1901957. doi: 10.1002/advs.201901957 (PMC7141037; doi:10.1002/advs.201901957)
Supplement: Supplementary file 1 — Supporting Information [file ADVS-7-1901957-s001.pdf]

Copyright WILEY-VCH Verlag GmbH & Co. KGaA, 69469 Weinheim, Germany, 2020.

Supporting Information

for *Adv. Sci.,* DOI: 10.1002/advs.201901957

Toward "On-Demand" Materials Synthesis and Scientific Discovery through Intelligent Robots

*Jiagen Li, Yuxiao Tu, Rulin Liu, Yihua Lu, and Xi Zhu**

Supporting Information

Towards the 'On-Demand' Materials Synthesis and Scientific Discovery through Intelligent Robots

*Jiagen Li, Yuxiao Tu, Rulin Liu, Yihua Lu, Xi Zhu**

Shenzhen Institute of Artificial Intelligence and Robotics for Society (AIRS), The Chinese University of Hong Kong, Shenzhen.

14-15F, Tower G2, Xinghe World, Rd Yabao, Longgang District, Shenzhen, Guangdong, 518172, China.

Email: zhuxi@cuhk.edu.cn

CONTENTS

S1. Details of MAOS

Figure. S1. Workflow of MAOS

S2. The Database

Table S1. The synthesis method of $CsPbBr_3$ QDs based on reprecipitation in method database

S3. The Pricing model

S4. Pseudocode of 'On-Demand' materials synthesis by reinforcement learning algorithms

S5. Communication test

Table S2. Summary of tested Ping value and bit rate through different mobile networks

S6. Hardware modules

Figure. S2. Scheme of compiling process in MAOS.

Figure. S3. Robot Arm Structure and Parameters

Figure. S4. VR devices

Figure. S5. Scheme and photograph of the reaction chamber in Chembox system

Figure. S6. Front side view of the reaction chamber in Chembox system

Figure. S7. Photograph and scheme of the heating module

Figure. S8. PL spectrum of Rhodamine B solution for temperature calibration

Figure. S9. Photograph of a working Photonic module

Figure. S10. Photograph and CAD model of the Liquid transfer module

Figure. S11. Photograph of the Cooling module

Figure. S12. Photograph of 3D Printer UltiMaker2+

S7. Experimental details for 'On-Demand' synthesis of CdSe QDs

Figure. S13. The estimated conditions and the FWHM difference where λ satisfy λ_t from 490 nm to 580 nm, $FWHM_t = 35$ nm.

S8. Other cases of 'On-Demand' synthesis by MAOS

Figure. S14. Scheme of automatic synthesis of PbS nanocrystals

Figure. S15. Spectrum (A) and TEM images of 'On-demand' synthesized PbS QDs.

Figure. S16. Temperature-dependent variation of first exciton absorption peak and PL wavelength of PbS QDs.

Figure. S17. Scheme of automatic synthesis of Au nanoparticles.

Figure. S18. Spectrum and TEM of synthesized Au nanoparticles.

Figure. S19. Scheme of automatic synthesis of MoO2 nanoparticles.

Fig. S20. The absorption spectrum of synthesized MoO_x nanoparticles.

Movie S1. The overall workflow of 'On-Demand' materials synthesis

S1. Details of MAOS

Workflow

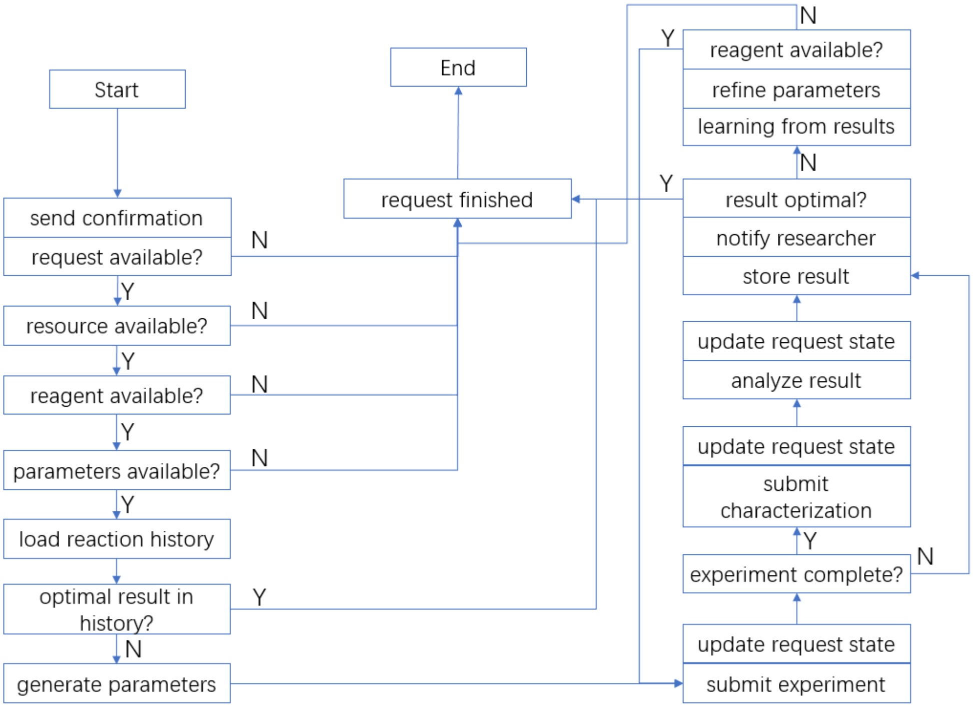

Figure. S1 The workflow of MAOS.

MAOS runs as a web server that receives synthesis request and optimizes the corresponding reaction parameters on the first-in-first-out (FIFO) principle. Figure. S1 shows the Workflow of how MAOS handling the request.

Environment Setup

1. **Prerequisites**

Hardware Requirements

- A powerful CPU

    - Laptop CPUs can often run the software, and the performance will influence software speeds

- **Our material synthesis hardware**
    - MAOS has hardware control interface, which controls all hardware and translates hardware-independent instructions into elementary basic operations.
    - Without our hardware, MAOS cannot communicate with hardware and raise exceptions.
- **Virtual reality head-mounted display (for VR software usage)**
    - HTC Vive

Supported operating systems

- MAOS could run on multiple Operating Systems including Windows 10, most Linux based distributions, and MacOS
- MAOS cores are implemented in Python, and the VR modules are implemented in Unity. Python and Unity are both cross-platform so that MAOS could run on the most mainstream operating system

2. Install Guide

Installing dependencies

- Python >= 3.5 64-bit
    - apt/yum install python3 (Linux)
    - Installer (Windows)
    - brew install python3 (MacOS)

  - Python 3 Anaconda environment is recommended, which could be download from https://www.anaconda.com/download/
- Unity >= 17.3 64-bit for VR environment
  - Download and install the latest Unity from https://unity.com/.
- MySQL (optional)
  - MAOS store long-term information through SQLAlchemy, which support multiple mainstream SQL database. MySQL, Postgres, Oracle, and SQLite are supported in MAOS and needs to be configured.
  - One of the databases must be configured, and MySQL is recommended.
  - MySQL could be downloaded from https://www.mysql.com/downloads/

Getting the MAOS code

MAOS can be directly downloaded from GitHub. For now, extract the code to a directory where you are comfortable working with it. Navigate to it with the command line.

Setup

Enter the folder that MAOS has been downloaded to and run the command to install Python dependent packages.

```
pip install -r requirements.txt
```

Notes

For now, this guide is far from complete. The new functionality may be updated over time, and new dependencies will be added and removed accordingly in the future.

3. MAOS Illustration

Overview

MAOS has multiple modules. These modules could run on a computer or multiple computers with internet access.

- **Hardware control interface:** Control all robotics hardware and handle all hardware dependent information
- **MAOS core:** Handle the request and optimize the reaction parameters to meet the demand
- **Human-machine interaction:** Provide multiple ways for users to interact with MAOS

Hardware control interface

Hardware control interface communicates with microcontrollers through serial and handles all hardware-dependent information. With our robotics hardware, the hardware control interface could work normally.

Run `cd hardware_controller & python hardware_control_interface.py`, input the port of each serial and a JSON-RPC based control interface would start on port 8888.

MAOS core

MAOS core is fully implemented in Python, which could handle the synthesis request, control hardware to do experiments through a hardware control interface, analyze the experiment results, and learn from the results. This module automates the synthesis reaction parameter optimization after a request. The request is implemented base on JSON-RPC protocol. Run python maos.py could start the MAOS core.

MAOS core includes optimizer and analyzer. For the learning policy of optimizer, an algorithm based on SnobFit is used currently. For analyzer, multi-peak gaussian wave fitting is implemented to analyze the spectrum data.

MAOS core needs to fetch and store data from the database management system (DBMS) so that the core could work normally. Run `python run.py to start MAOS core. The request handler would start on port 9999.`

Human-machine interaction

- **UI:** A simple UI implemented with PyQt is provided in `\human_machine_interaction\UI`, which could support low-level control of the hardware. Currently, syringe pump control is supported. Open the UI by running `cd human_machine_interaction\UI & python ui.py`

- **VR:** A VR software is implemented in Unity. Open `\human_machine_interaction\VR\Assets\Scenes\SampleScene.unity` in Unity, and you can control robot arm virtually. The control of the real robot arm needs our robot hardware and the communication with the hardware control interface.

- **Website:** A basic version website where the user could submit synthesis demand is implemented in Flask. The web server would start on port 80 after run `cd human_machine_interaction\website-basic & python run.py`. This website needs to connect the database with material and synthesis data. MAOS core should be working when submitting the request.

Database Config

MAOS fetch and store important information from database through SQLAlchemy. So, MAOS supports multiple mainstream SQL database such as MySQL, Postgres, Oracle, and SQLite. `maos\__init__.py` records the database config URI. This database connection

URI should be modified (https://flask-sqlalchemy.palletsprojects.com/en/2.x/config/#connection-uri-format) to configure the database.

After configuration, run `cd scripts & python create_db.py` to initialize the database table and insert little test data. Currently, we do not upload all the data in our database.

MCU

- **Arduino:** An Arduino Uno board is used to control a stepper motor and two gas valves. MsTimer2 is used to control the time accurately.
- **STM32:** A STM32 F103 development board is used to control the peristaltic pumps and a stepper motor.

Notes

It is the first version of MAOS; more functionalities are under construction.

S2. The Database

Materials property database: This database is built up with online open source such as Open Quantum Materials Database (OQMD) [1]. Properties such as bandgap, space group id, the magnetic moment of 66993 kinds of inorganic materials are collected.

Regent database: We sort out 1052 reagent information based on our purchase records. Information includes purity, concentration, packing size, and price.

Method database: The method database is constructed with materials synthesis methods detailed with parameters in each step. So far, solution-based methods for various kinds of

nanomaterials synthesis including CdSe, CdS, PbS nanocrystal for optoelectronic devices, Au and MoO_2 nanoparticles for plasmonic photothermal applications. An example here is the synthesis method of $CsPbBr_3$ QDs in Table S1.

Table S1. The synthesis method of $CsPbBr_3$ QDs based on reprecipitation in method database

	**Reagent**	**Amount**	**Condition**	**Time**
**Step 1**	CsBr	0.4 mmol	Stir 300RPM, 25□	10min
	PbBr2	0.4 mmol		
	DMF	10 ml		
**Step 2**	OA	1ml	Stir 300RPM, 25□	3min
	OM	0.5ml	Out P1	
**Step 3**	P-xyline	20ml	Stir 500RPM, 25□	5min
	P1	1ml	Out	

Machine database: This database records the type of hardware we support now and some hardware parameters. It helps MAOS to judge the experiments our hardware could do and the maximum result space it could search. Details of modules in this database are in supporting information S4.

Result database: All the experiment results would be stored in the result database. The amount of data represents how well we know about the reaction. The history experiment results significantly accelerate the optimization for a different target.

S3. The pricing model

Nowadays, the concepts of materials synthesis are not only the reaction of matters, collecting or breaking of chemical bonds, but a deep fusion with the environment, economics, and human life. The evaluation of synthesis experiments should be treated as a hybrid model. Here, to encourage researchers to approach the design principle of "green chemistry" [2], a *pricing model* of MAOS is constructed for evaluating the total cost C_{total} of each experiment. It is an overall evaluation of recourse expending including three issues: chemical reagent, materials toxicity, and local prices, forming as

$$C_{total} = (C_{reagent} + C_{toxicity}) * I_{prices}$$

The cost of chemical reagent $C_{reagent}$ is calculated according to the reagent database, which contains the price information of all purchased reagent in the lab.

$C_{toxicity}$ is evaluated according to the toxicity and pollution strength of the materials synthesized by the reagents, and the by-products during experiments. This cost is set to

encourage researchers to optimize synthesis methods and generate materials with little or no toxicity to human and environment. The identification of $C_{toxicity}$ is based on Environmental Protection Tax Law [3] promulgated at the 25th meeting of the 12th Standing Committee of the National People's Congress. $C_{toxicity}$ can be calculated with:

$$C_{toxicity} = toxicity\ equivalent\ value * mass(kg) * tax\ rates$$

Here, $toxicity\ equivalent\ value$ is the scale of tax for different kinds of materials with unit mass. For example, Cd-compound is 0.005RMB/kg, and Pb-compound is 0.025RMB/kg, P-xylene is 0.02RMB/kg, details can be found in the *Table of Taxable Items and Tax Rates of Environmental Protection Tax* [3]. Tax rates are related to the local government of lab.

I_{prices} is evaluated according to the local prices of the lab, which is highly location-dependent. The cities with high infrastructure and workforce cost will have large I_{prices}. Here we introduce the Global City Index (GCI) [4] to evaluate I_{prices} in following the format:

$$I_{prices} = GCI$$

We collected the cost of $CsPbBr_3$ QDs synthesis in Hong Kong and Shanghai with same production mass to test I_{price} in this model. It showed that the total cost in Hong Kong is 1.33 times of that in Shanghai, which is close to the ratio of GCI (1.41) of these two cities.

S4. Pseudocode of "On-Demand' materials synthesis by reinforcement learning algorithms

```
function HANDLE_REQUEST (request_id)
        target, reaction_id ← FETCH_REQUEST_INFO
(request_id)
        config ← FETCH _REACTION_CONFIG (reaction_id)
        if not CHECK_RESOURCES (reaction_id) then
                raise Exception
        end if
        reaction ← GET_REACTION (config)
        optimizer ← GET_OPTIMIZER (config)
        reaction.initialize()
        optimizer.initialize()
        LOAD_REACTION_HISTORY (optimizer, reaction_id)
        if OPTIMAL (optimizer.get_best()) then
                return optimizer.get_best()
        end if
        n_call←0
        while n_call < config.max_call do
                action ← optimizer.choose_action()
```

```
                result ← reaction.experiment(action)
                reward ←
reaction.analyzer.calc_reward(result, target)
                STORE_RESULT (action, result)
                optimizer.update_env(action, reward)
                n_call ← n_call + LENGTH(action)
                if OPTIMAL (optimizer.get_best()) then
                        return optimizer.get_best()
                end if
        end while
        return optimizer.get_best()
end function
```

Notes:

FETCH_REQUEST_INFO:

load request information from the database.

FETCH _REACTION_CONFIG:

loads the configure file of the reaction from the database, which contains all the information about how to synthesis and the hyper-parameter of the optimizer.

CHECK_RESOURCES :

checks if the hardware works well and the reagent is enough.

The reaction in charge of communicating with the hardware control interface and controlling the experiment optimizer choose the reaction parameters (action) and learn from the analysis result (reward).

LOAD_REACTION_HISTORY:

load all experiments about this action and update the state of the optimizer.

STORE_RESULT:

store the experiment results in the database.

S5. Communication test

Higher data rate and lower delay are eager for collaborative robots and real-time system. The burden is sometimes a big cable for flexible applications. Antenna based mobile networks are proved efficient in robotic automation. 5G technology is regarded as the revolution power for future robotic industry. Here, we tested the performance of three different generations of networks collecting MAOS and mobile devices (Shown in table S1). Under 5G (provided by China Unicom & Ericsson), 385Mbps data rate enables real-time monitoring of 90-120fps video flow with high resolution (1920*1080) captured by an industrial camera. 22ms Ping also significantly enhanced the preciseness of remote control of robot cells.

Table S2. Summary of tested Ping value and bit rate through different mobile networks

	**3G**	**4G**	**5G**
**Ping**	140 ms	80ms	22ms
**Transmission rate**	9Mbps	75Mbps	385 Mbps

S6. Hardware Modules

Compile

An example of compiling the input experimental formula to machine instruction code is shown in Figure S2. This example shows how MAOS extracts information from input formula, and set up all experimental parameters in configuration file for CdSe QDs synthesis. The configuration file was generated through designed template. It decides the all adjustable or constant parameters for optimizing the experiment.

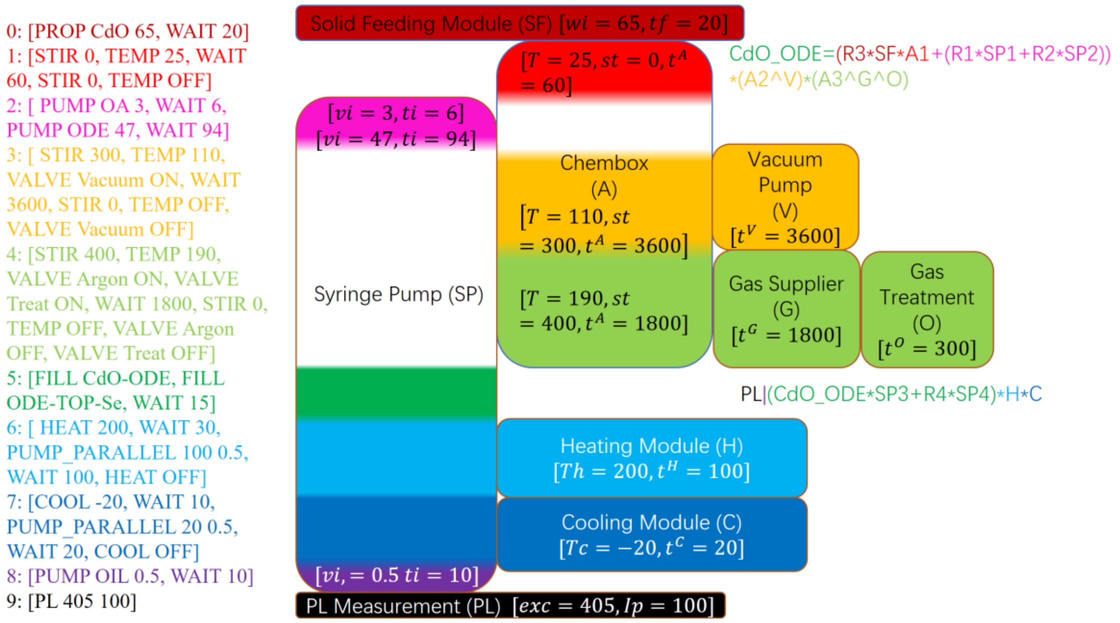

Figure. S2. Scheme of compiling process in MAOS. The color if icons and cods indicate different steps in experiment. Firstly, the formula (on right) with vectors of module parameters (in each icon) were input to MAOS. Then, the instruction codes (on left) were generated through compiling process.

Configuration file for 'On-Demand' synthesis of CdSe QDs.

{"type": "fluid reaction",

"name": "CdSe Quantum Dots",

"reagent": [{"formula": "CdO-ODE-Precursor", "ratio": 1D,

{"formula": "Se-Top-ODE-Precursor", "ratio": 1}],

"metric": ["wavelength", "LFWHM"],

"param": {"temp": {"default": 40, "bound": [170, 245], "dx": 5, "var": 1}figfifi,

“Se-Top-ODE-Precursor”: {“default”: 1, “bound”: [0.001, 1000.0], “dx”: 0},
“time”: {“default”: 30, “bound”: [10, 600], “dx”: 3, “var”: 1}},
“init”: [“PUMP_INIT”],
“experiment”: [[“FILL CdO-ODE”, “FILL ODE-TOP-Se”, “WAIT 15”],
[“HEAT $temp”,
“WAIT 30”,
“PUMP_PARALLEL $time 0.5”,
“WAIT $time”,
“HEAT OFF”],
[“COOL -20”, “WAIT 10”, “PUMP_PARALLEL 20 0.5”, “WAIT 20”, “COOL OFF”],
[“PUMP OIL 0.5”, “WAIT 10”],
[“PL 405 100”]],
“optimizer”: “snobfit”,
“max_expts”: 100,
“nreq”: 0}

Collaborative robot

a

b

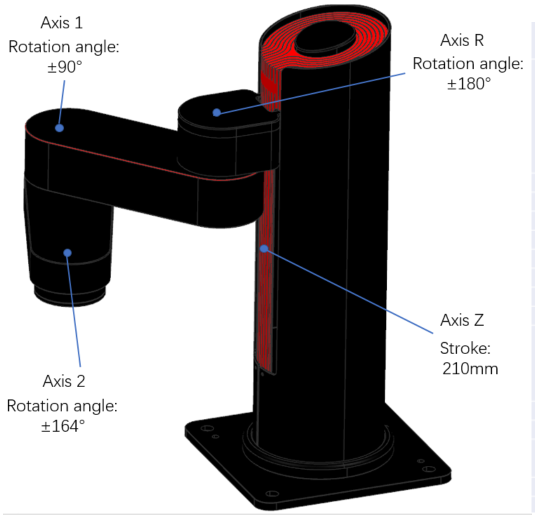

Parameter			Model
			Z-Arm 2140 Non-cooperative
Basic Information	Axis 1	Arm Length	200mm
		Rotation Angle	±90°
	Axis 2	Arm Length	200mm
		Rotation Angle	±164°
	Axis Z	Stroke	210mm
	Axis R	Rotation Range	±180°
Line Speed			1255.45mm/s(1.5kg load)
Repeatability			±0.03mm
Standard Load			1.5kg
Maximum Load			2.5kg
Degree of Freedom			4
Power			220V/110V 50~60Hz adapt to 24V DC
Communication			WiFi/Ethernet
Expandability			Built-in Integrated Motion ,offer 10I/O ports
I/O port	Digital Input(isolated)		5
	Digital Output(isolated)		5
	Analog Input(4-20mA)		/
	Analog Output(4-21mA)		/
Machine Height			558mm
Host Weight			19kg
Base Mounting Size	Base Dimensions		250mm*250mm*10
	Base Fixing Hole Spacing		200mm*200mm with 4 screws of M8*20
Impact Checking			×
Drag and Teach			×

c

d

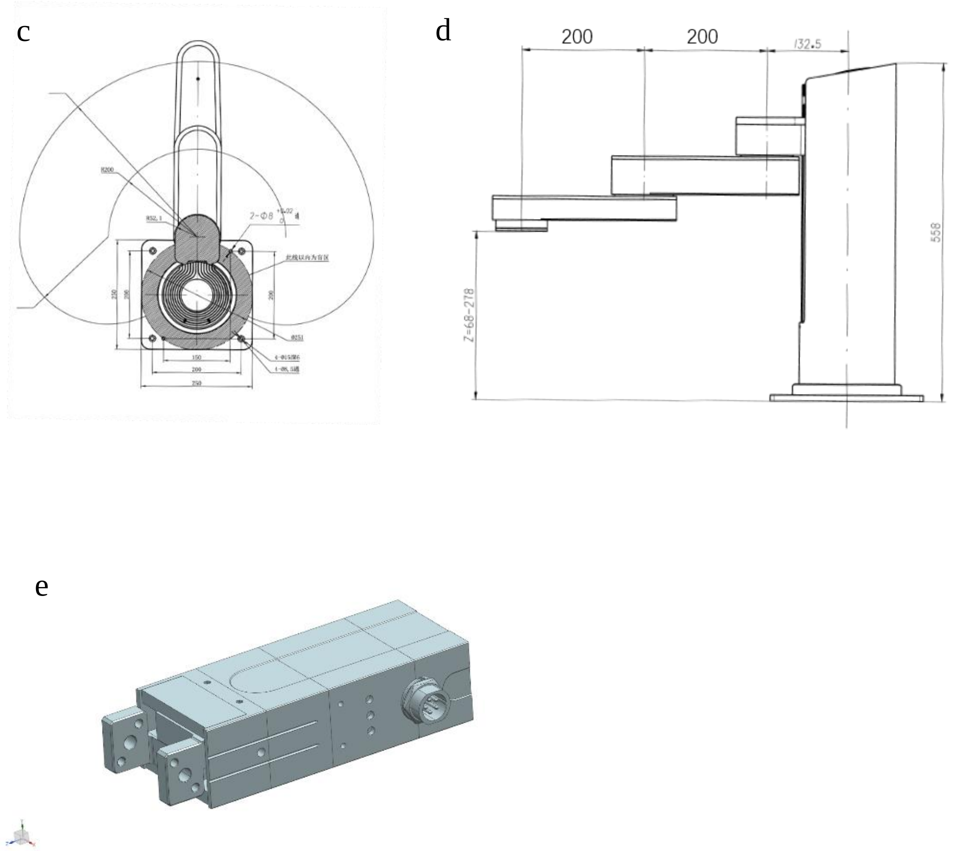

e

Figure. S3. Robot Arm Structure and Parameters. **a** The appearance of a collaborative robot and range of joint motion. **b** Collaborative robot hardware parameters. **c** Range of motion of the collaborative robot. **d** Arm length of collaborative robot. **e** The appearance of end effector (grasper).

As shown in Figure. S3, the collaborative robot has four degrees of freedoms. An end grasper is equipped for transfer the reagent containers.

VR devices

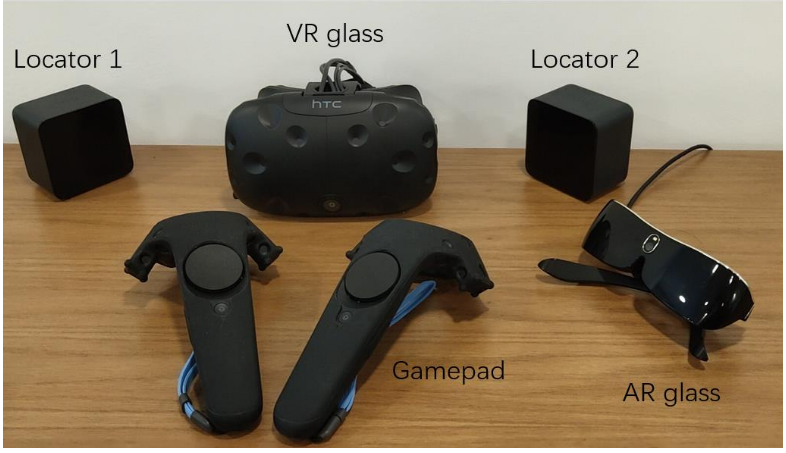

Figure. S4 HTC Vive and our AR glass.

As shown in Figure. S4, HTC Vive suite, including a VR glass, two gamepads, and two locators are utilized for remote control of the collaborative robot. An AR glass was used to share the live vision in HTC Vive glass.

Chembox

The chamber in Chembox provided a milliliter scale reaction system integrated with multiple functional devices, including temperature, magnetic stir, and in-situ computer vision monitoring. The feeding system combined on reaction chamber of Chembox system (shown in Figure. S5) can fit the requirement to control the injection of both liquid and solid reactants. The solid sample is stored in a horizontal arranged tube; the push rod can move on the screw rod and motor three control the speed and distance. The push rod is used to push the powder in the tube to the reaction vessel. The whole feeding system can move on the sideway

controlled by the motor. A quadrantal half-cylinder-shaped tube fabricated by the 3D printer was used for liquid sample and connected with a peristaltic pump via a rubber tube. All feeding system was controlled by an STM32 chip on top. In this work, we made an upgraded by integrating an automatic weighing system for Chembox system. As shown in Figure. S6, the total mass of added reactant, solution volume, and reaction condition information can be displayed on an LCD interface. Solenoid valves controlled by Arduino chip were utilized to provide vacuum or N2 atmosphere environment for a specific reaction.

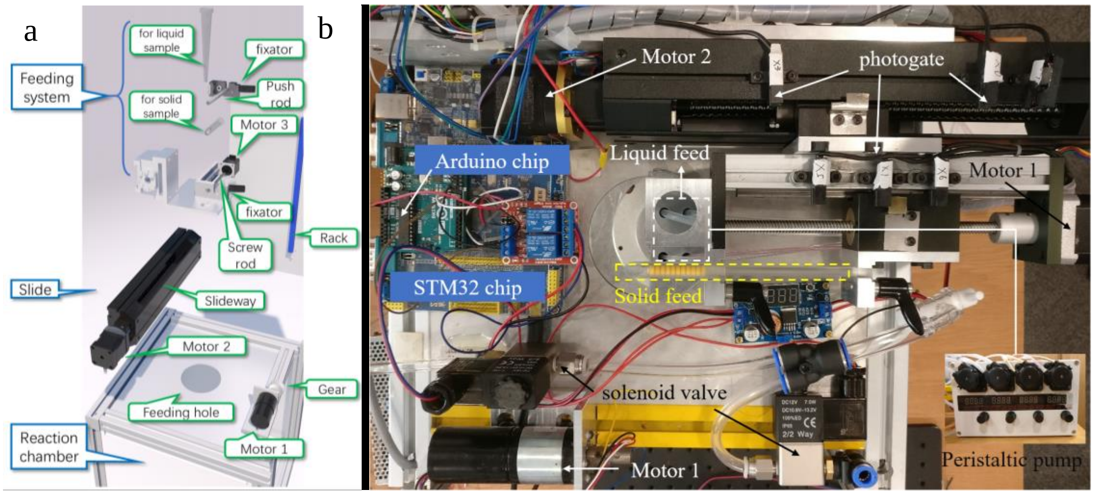

Figure. S5 Scheme and photograph of the reaction chamber in Chembox system. **a** Detailed scheme of each component of the system bonded on the reaction chamber. **b** Photograph from downside view of the reaction chamber.

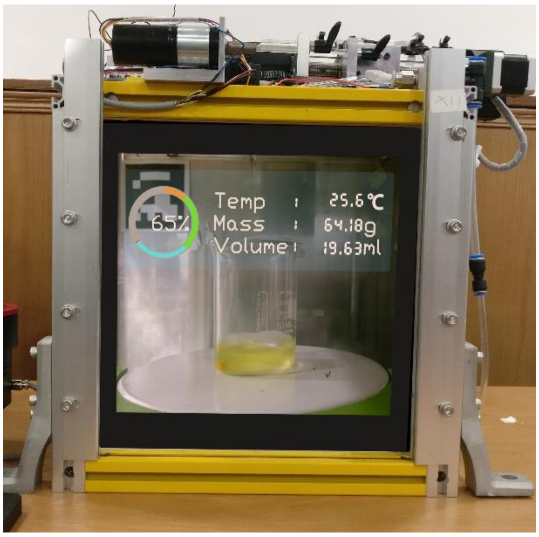

Figure. S6 Front Side View of Reaction Chamber. A tablet is fixed on the front of the reaction chamber. The screen shows the camera view which on the back of the tablet. The reaction information shown on the interface is fetched from sensors.

Heating Module

As shown in Figure. S7, the heating module was made up with a copper pipe (OD=20mm, L=150mm), an electric heating cartridge, a temperature sensor，a PID controller and position stages. PTFE tube was coiled and sat within a groove with a 1mm radius engraved on the surface of the copper pipe. The heating cartridge (D=14mm, L=150mm, Max power=100W) was embedded in copper pipe. The real temperature was monitored by a patch K-type thermocouple (-100℃-350℃) with 0.1℃ accuracy, embedded between PTFE tube and cooper. The temperature was controlled by a PID controller, with a temperature variation below ±1℃ after 5 minutes warm-up. The PID device was controlled through serial communication by STM32 microchip.

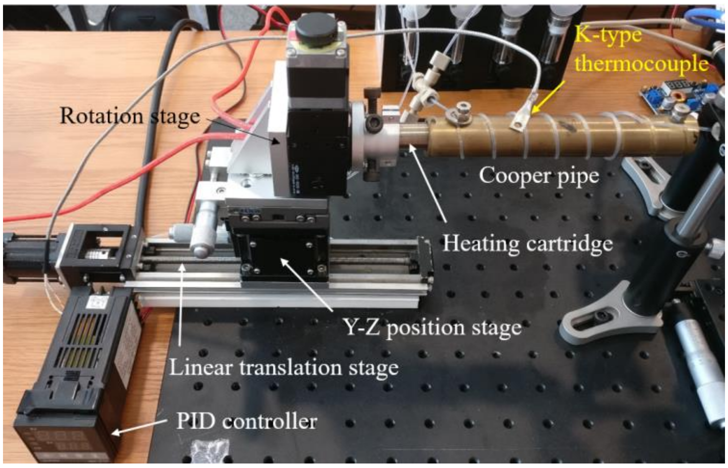

Figure. S7 Photograph and scheme of the Heating module. The Heating module combined heating cartridge, temperature controller, and motored position stages provides stable temperature from room temperature up to 350 to heat reactions flow in the coiled microtube. 4-axis stages allow precise position adjusting in multiple directions.

Temperature calibration. Temperature calibration of chemical flow in heat zone was designed according to Kovalenko and deMello 's work in 2016 [5]. It utilized the decrease of fluorescence intensity of Rhodamine B continuously with increasing the temperature [6]. The PL spectrum of Rhodamine B in water (100 µM) at a different position in optimizes heating zone (corresponding to different heating time) was collected in Figure. S8. PL intensity with under 1% variation among three-time points (1s, 5s, 25s), which quantitatively verified the fast heating efficiency of this Heating module.

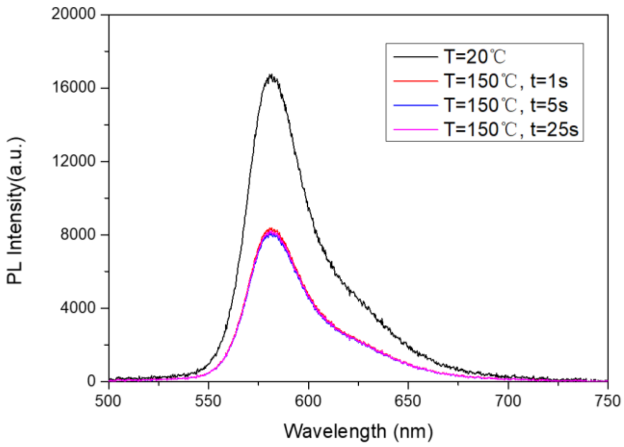

Figure. S8 PL spectrum of 100 µM Rhodamine B at 25 °C and 150 °C for temperature calibration. The red, blue and pink line represents the PL spectrum of Rhodamine B solution entering the Heating module at 1s, 5s, and 25s, separately. The nearly same PL intensity proved the fast heating ability of reaction flow within 1 second.

Photonic Module

The Photonic module was made up of 120 UV LEDs (395nm emission wavelength) integrated on the soft electronic strip and boned in the tube (Figure. S9). Power of each LED is 0.25W, and the total power is 30W. The 3D-printed tube has an 8cm diameter and 12cm length, which allows the copper pipe suspended in and receive UV radiation. DC voltage source controlled through serial communication can adjust supply voltage from 0V to 12V. Different waveform, including square wave, saw-toothed, and sin-cos wave can be generated. The working stability of these LEDs was determined under 50℃ for 24 hours. The max radiation power slightly decays to 97% to the initial state.

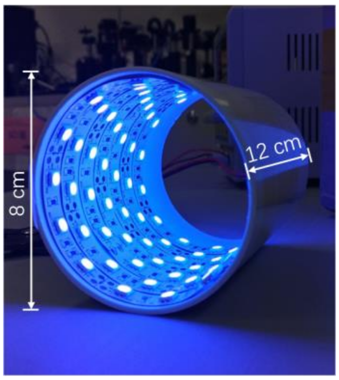

Figure. S9 Photograph of a working Photonic module. 120 UV LEDs (395nm wavelength, 0.25W for each) were integrated for investigation of photon-induced reactions.

Liquid transfer module

A transfer module was designed as a connection center for different synthesis and characterization modules (Figure. S10). An up-down system made up with a stepping motor

and screw rod was assembled for guiding the tube in-out of solution container. The tube guider was manufactured by 3D printer with PLA materials.

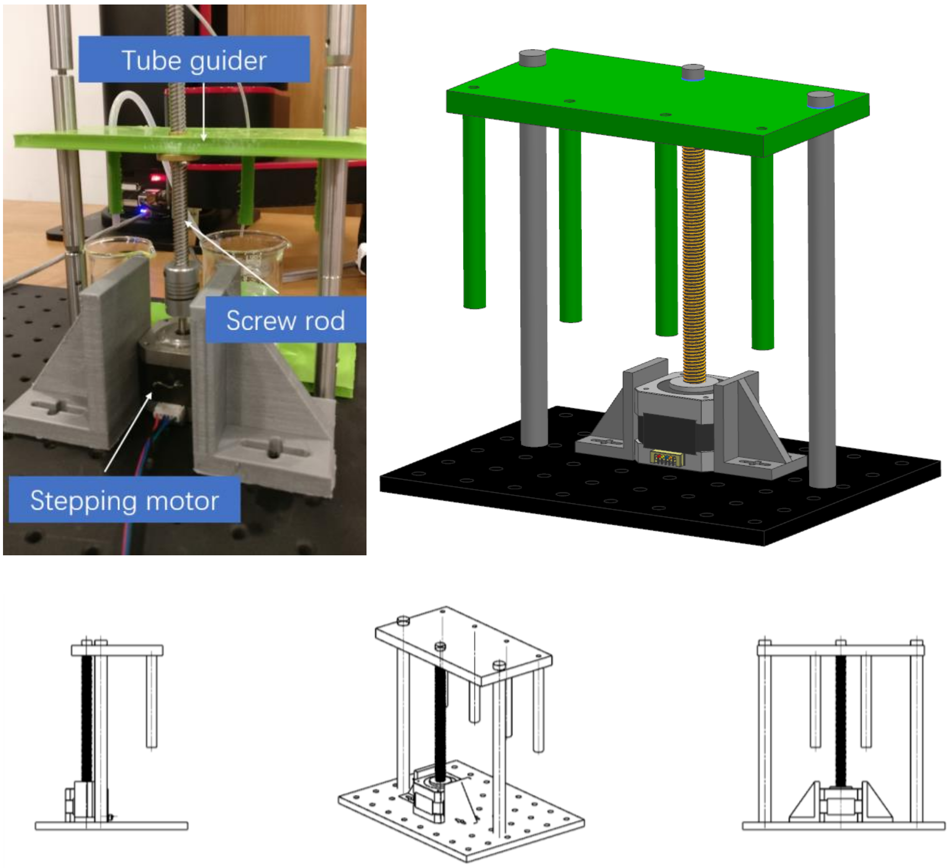

Figure. S10 Photograph and CAD model of the Liquid transfer module. The up-down system enables a speed range from 1mm/s to 5mm/s. The 3D-printed tube guide was designed to collect different solution container and microfluidics.

Cooling module

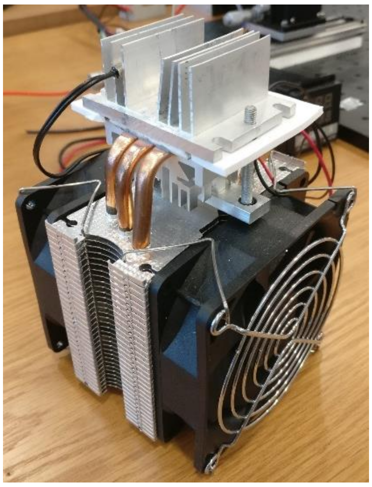

Figure. S11 Photograph of Cooling module.

The Cooling module was designed based on a semiconductor refrigerator (75W) and air fans (Figure. S11). Serials communication was applied to adjust the local temperature from -20℃ to room temperature.

3D-Printer

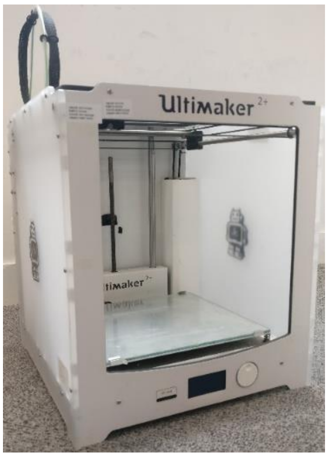

Figure. S12 Photograph of 3D Printer UltiMaker2+ (maximum print size is 223×223×205mm).

As shown in Figure. S12, the 3D Printer is used to print machine parts.

S7. Experimental details for the 'On-Demand' synthesis of CdSe QDs

Materials

Cadmium oxide (CdO, Sigma-Aldrich, 99.99%), selenium (Se, 99.99% Sigma-Aldrich), oleic acid (OA, Sigma-Aldrich, 90%), 1-octadecene (ODE, Sigmal-Aldrich, 90%), trioctylphosphine (TOP, Sigma-Aldrich, 97%)

Precursors preparation and microfluidic synthesis of CdSe QDs followed Bawendi's work [7].

Cd Precursor (5mM) was prepared in a 100 mL round-bottom flask. 65 mg of CdO and 3 mL of oleic acid (OA) were added. 47 mL of ODE was then injected through syringe pumps. Vacuum environment was applied to degas for 30 minutes at room temperature and then degassed under vacuum at 110 °C for 1 h. The mixture was then sufficiently dissolved under an argon atmosphere at 190 °C and 400 rpm stirring for 30 minutes.

Se Precursor (10 mM) was prepared in a glove box by mixing 80 mg Se powder, 5 mL TOP and 45ml ODE in a 100ml bottle. After stirring at 400rpm for 3h at room temperature, the mixture became a colorless solution.

Microfluidics synthesis of CdSe QDs. OEM (Original Equipment Manufacturer) syringe pumps (MSP1-C2, Longer, China) were used to inject the dispersed phase (Cd precursors and Se precursor solutions) towards a polyether ether ketone (PEEK) cross-junction to mix and into the Heating module. 0.5ml syringes were used here. The injection cross-junction and the syringes carrying the precursor solutions were connected through PTFE tubing (ID 1/32'', OD 1/16'') using PEEK finger-tight fittings. The mixed precursor solution flows through PTFE tubing (ID 1/32'', OD 1/16'') coiled around Heating module. The reaction time was controlled by varying the total flow rates of two kinds of precursors. Temperature range controlled by the Heating module was from room temperature to 235℃.

Results of optimized solutions for various target wavelengths

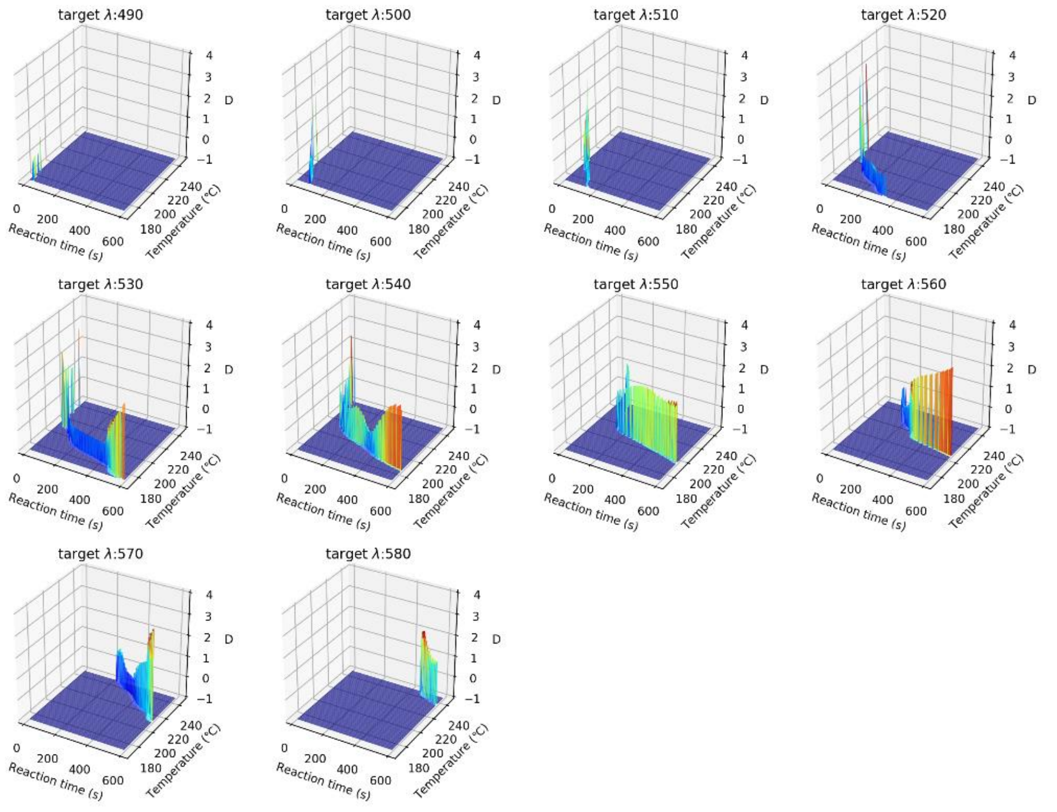

Figure. S13 The estimated conditions and the FWHM difference where λ satisfy λ_t from 490 nm to 580 nm, $FWHM_t = 35$ nm. D represents the FWHM difference, which is defined as

$$D = \begin{cases} ReLu(FWHM - FWHM_t), & |\lambda - \lambda_t| < 0.5 \\ -1, & otherwise \end{cases}.$$

Shown in Figure. S13, ten pictures collected original data of all optimized parameter sets which satisfy user demand of λ_t. The information of FWHM was indicated by parameter D.

Details of multiple injections of precursors

In the optimization process of the size distribution of nanoparticles, according to the result derived by Clark[8], the average radius $\langle r \rangle$ and the standard deviation σ in ordinary growth condition could be tuned by adjusting the concentration of the monomers via the relation

$$\frac{\sigma(t)}{\sigma_0} = \left(\frac{\langle r \rangle}{\langle r \rangle_0}\right)^{\frac{1}{\xi}-1}, \#Eq(S7.1)$$

where $\xi = \frac{v_m \dot{c}}{4\pi Z} \frac{1}{K_D} \propto \dot{c}, K_D = \frac{2\gamma v_m^2 c_\infty D}{RT}, r_c = \frac{2\gamma v_m c_\infty}{RT(c-c_\infty)}$, v_m is the molar volume of the material, $\dot{c}$ is the time derivative for the concentration of monomers, Z is the total amount of nucleated material in all the nanoparticles, γ is the surface potential, c_∞ is the bulk concentration of the considered material, D is the diffusion coefficient, r_c is the critical radius under certain concentration c, R, T are the gas constant and temperature.

In the work present, the scenario to be optimized is to improve the size distribution of the nanoparticles produced with a fixed amount of monomers injected. Inspired by the work of Alivisatos[9], the optimization process was done by separately injecting the fixed amount in several times (defined as η), each separated by a time interval (defined as τ). Thus,

$$\xi \propto \sum_{n=0}^{\eta} \Theta(n\tau)\, exp\left(-\frac{t-n\tau}{\mu}\right). \#Eq(S7.2)$$

Thus, right after the k-th injection and before the $(k+1)$-th injection,

$$\xi = \xi_0 exp\left(-\frac{t}{\mu}\right)\left(1 - exp\left(\frac{k\tau}{\mu}\right)\right)\left(1 - exp\left(\frac{\tau}{\mu}\right)\right)^{-1}. \#Eq(S7.3)$$

T is the total reaction time, μ is the parameter used to scale the reaction speed, which is assumed to be constant. The function Θ is the Heaviside Theta function, which takes only 1 at positive numbers, and zero elsewhere used to resemble the discrete injection. By defining such a $\xi \propto \dot{c}$, i.e., concentration, since $\frac{d}{dt}\langle R \rangle = \int_0^\infty f \frac{dR}{dt} dR = \left\langle \frac{2\gamma v_m^2 c_\infty D k}{(D+kR)KT}\left(\left(\frac{2\gamma v_m c_\infty}{c-c_\infty}\right)^{-1} - \frac{1}{R}\right)\right\rangle$ in ordinary growth and $\langle R \rangle = R^* = R_0^*\left(1 + \frac{2\gamma D c_\infty v_m^2}{r_0^* KT(9/4)}\right)$ during Ostwald Ripening by Wagner[10], $\langle R \rangle$ could be numerically calculated based on the $c(t)$ given above with $\langle R \rangle_0$. Thus,

$$\frac{\sigma_t}{\sigma_0} = \left(\frac{\langle R\rangle_t}{\langle R\rangle_0}\right)^{\frac{1}{\xi_t}-1} = \left(\frac{\langle R\rangle_t}{\langle R\rangle_0}\right)^{\frac{1}{\xi_0 exp\left(-\frac{t}{\mu}\right)\left(1-exp\left(\frac{k\tau}{\mu}\right)\right)\left(1-exp\left(\frac{\tau}{\mu}\right)\right)^{-1}}-1} \#Eq(S7.3)$$

if the time t was just right after the k-th injection and before the $(k+1)$-th injection.

By such an expression of σ_t, it could be proved that there must be intersections between different schemes. It could be proved from the existence of an intersection in σ between the two extreme cases: $\eta \to \infty$ and $\eta = 1$. While $\eta \to \infty$ resembles constant concentration, $\eta = 1$ resembles the most fundamental case, in which the reaction takes place all at once. That is to prove

$$\left(\frac{\langle R\rangle_T^{\eta=1}}{\langle R\rangle_0}\right)^{\frac{1}{\xi_T^{\eta=1}}-1} > \left(\frac{\langle R\rangle_T^{\eta\to\infty}}{\langle R\rangle_0}\right)^{\frac{1}{\xi_T^{\eta\to\infty}}-1}, \#Eq(S7.4)$$

$Eq(S7.4)$ can be proved by referring to the fact that $\langle R\rangle_T^{\eta=1} > \langle R\rangle_T^{\eta\to\infty}$, and by the definition of $c(t)$ above, $0 \approx \xi_T^{\eta=1} < \xi_T^{\eta\to\infty}$, both positive and close to zero. Thus, Equation (S7.4) holds, indicating possibilities for optimization of the reaction scheme. Note that $\tau \to 0$ is definitely not the best option always.

S8. Other Cases of 'On-Demand' Synthesis by MAOS

So far, various kinds of solution-based nanomaterials synthesis for different application situations were covered. CdSe, PbS nanocrystals were synthesized for optoelectronic devices and Au and MoO_2 nanoparticles for photothermal applications.

1. PbS nanocrystals

We provide PbS nanocrystal for the demand of near-infrared optoelectronic devices. A hot-injection synthesis strategy is used for PbS nanocrystal synthesis [11]. Scheme of automatic synthesis process is shown in Figure. S14. 1.1g (220 mg × 5) of PbO was first loaded into 100ml three-necked flask in Chembox. Then 3.25 mL of oleic acid (OA), 20 mL of 1-octadecene (ODE) was injected. Stirring the mixture at 300rpm and pump to 200mTorr. Then, temperature set to 150℃ until solution color changes from yellow to clear (10 minutes). Finally, change the temperature to 120℃ and keep the N_2 environment by flowing N_2 gas (10 minutes). 0.5ml of TMS was rapidly injected (1ml/s) into Chembox. After 3 minutes reaction, the solution was extracted through cooling, Abs, and PL module.

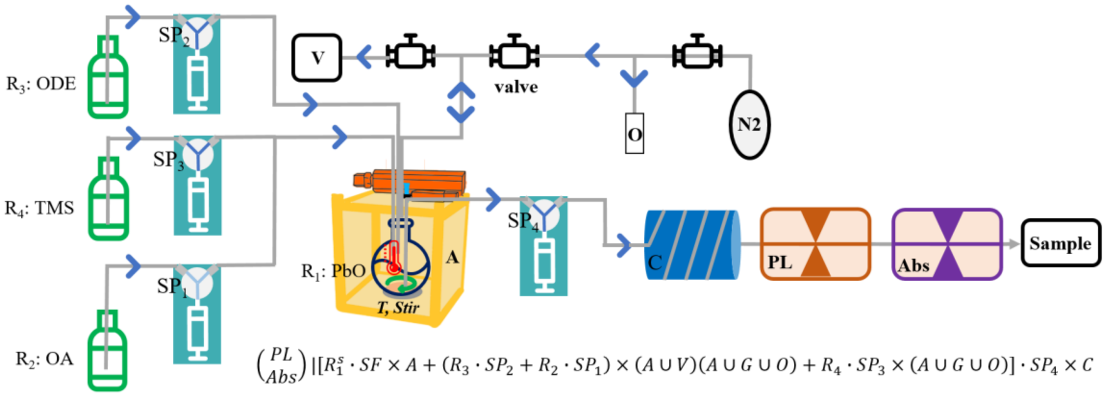

Figure. S14 Scheme of automatic synthesis of PbS nanocrystals

Photoluminescence properties of PbS nanocrystal synthesized with different temperature was collected, shown in Figure. S15a. The TEM image (Figure. S15b-d) shows that PbS QDs are well single-dispersed, and the size increases with the rising of temperature. As a result, the wavelength of first exciton absorption peak and PL peak both went through a redshift. The scaling of temperature-dependent optical properties of PbS QDs is shown in Figure. S16. With this scaling, 'On-Demand' synthesis of PbS QDs with target emission wavelength from 900nm to 1450nm can be well handled with MAOS.

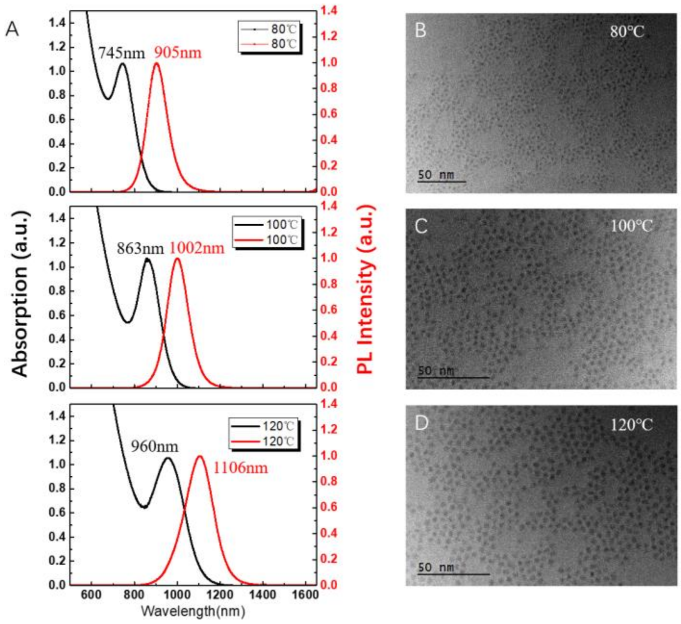

Figure. S15 Spectrum and TEM images of 'On-Demand' synthesized PbS QDs.

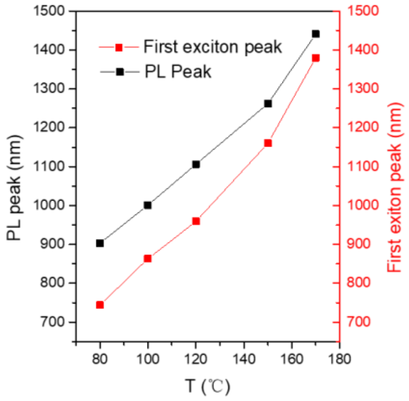

Figure. S16 Temperature-dependent variation of first exciton absorption peak and PL wavelength of PbS QDs.

2. Au nanoparticle

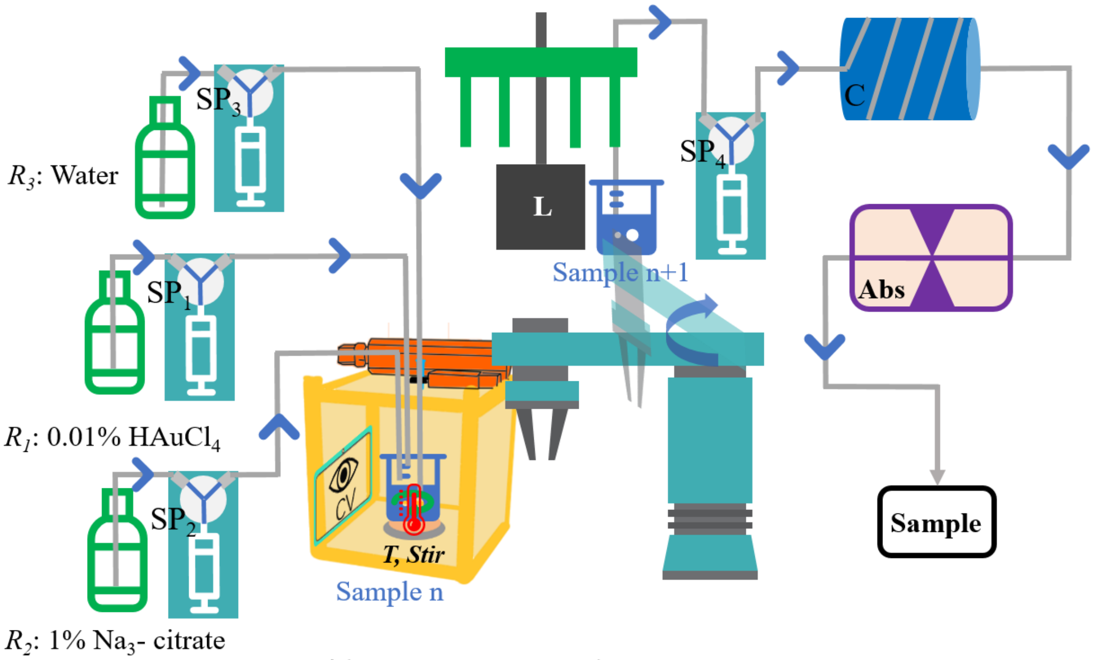

$$CV|(R_1 \cdot SP_1 + R_2 \cdot SP_2) \times A + R_3 \cdot SP_3 \times A$$

$$Abs|[(R_1 \cdot SP_1 + R_2 \cdot SP_2) \times A + R_3 \cdot SP_3 \times A] \cdot CR \cdot L \cdot SP_4 \times C \times Abs$$

Figure. S17 Scheme of automatic synthesis of Au nanoparticles

Gold nanoparticles with tunable plasmonic properties are widely used for fields of catalytic, photothermal and bioimaging. Here, MAOS was applied for the ‘On-Demand’ synthesis of Au nanoparticle with precisely controlled size and narrow size distribution. The method is based on the reduction of gold chloride with sodium citrate [12]. Pure water is used to adjust the concentration of liquid reagents. CV module here was set to monitor the value and color information of reaction. Absorption of products will be measured with the help of the robot and liquid transfer module.

According to the reference, with the various ratio of Na_3-citrate : $HAuCl_4$, nanoparticles with different size can be synthesized. However, the problem of reproducibility caused by experimental environment sometimes results in a diameter variance, and even worse, poor quality. MAOS needs a random searching process through absorption spectrum and then

benchmarked with the data in reference. 15 random parameter set contains different Na_3-citrate : $HAuCl_4$ ratio was first implemented. Figure. S17a shows the spectrum of 9 samples with measurable absorption peak. The samples with the highest absorption peaks (violet and green line) were processed for TEM characterization (Figure. S17b-c). Thus, the recipe for Au nanoparticles with 5nm and 11nm average size were found, and the mapping of size-ratio was updated based on these experimental results. With this practical mapping, ‘On-Demand’ synthesis of Au nanoparticles with 5nm, 8nm, 10nm, and 15nm and narrow size distribution were synthesized (Figure. S17 d-g).

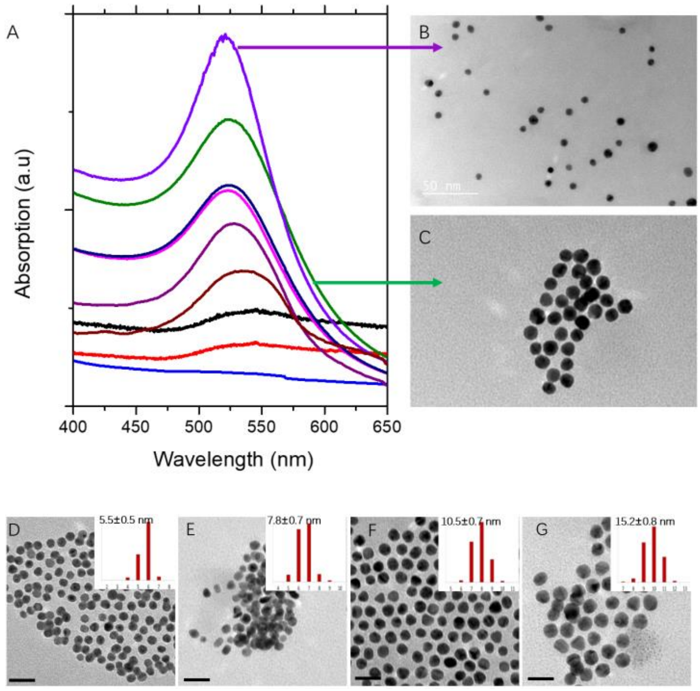

Figure. S18 Spectrum and TEM of synthesized Au nanoparticles. **a-c** Benchmark process through spectrum screening and a few shots of TEM. The scale bar in B, C, is 50nm. **d-g** ‘On-Demand’ synthesized Au nanoparticles with target size and size distribution. The scale bar is 20nm.

3. MoO_2 nanoparticles

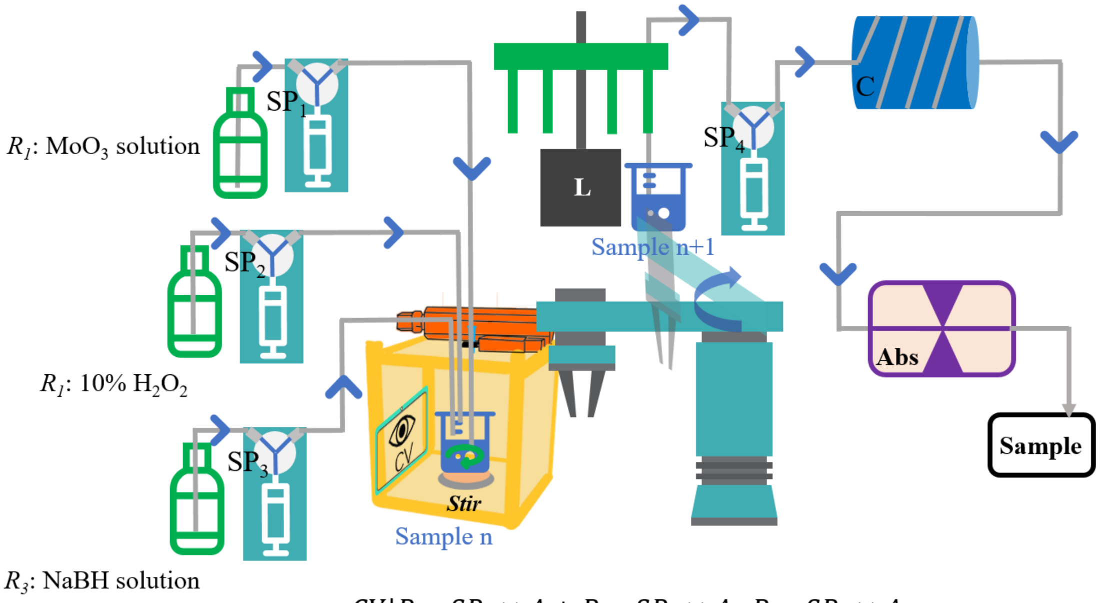

$$CV|R_1 \cdot SP_1 \times A + R_2 \cdot SP_2 \times A + R_3 \cdot SP_3 \times A$$

$$Abs\ |(R_1 \cdot SP_1 \times A + R_2 \cdot SP_2 \times A + R_3 \cdot SP_3 \times A) \cdot CR \cdot L \cdot SP_4 \times C \times Abs$$

Figure. S19 Scheme of automatic synthesis of MoO2 nanoparticles.

Similar to Au nanoparticles, MoO_2 nanoparticle has easily adjustable plasmonic properties and practical synthesis method [13]. By adding different amount of oxidant (H_2O_2 solution) and reductant (NaBH solution), (Figure. S19) intensity of the plasmonic peak of MoO_x (x various from 3 to 2) changes. Figure. S20 shows that most substantial plasmonic peak appears when NaBH:Mo=2. The strong plasmonic signal at near 800nm can satisfy the demand of NIR bio imaging.

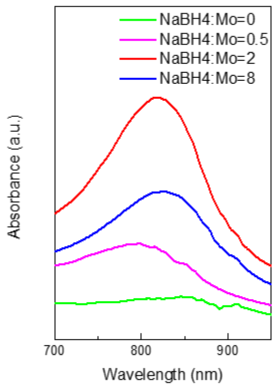

Figure. S20 The absorption spectrum of synthesized MoO_x nanoparticles.

Reference

[1] S. Kirklin, J. E. Saal, B. Meredig, A. Thompson, J. W. Doak, M. Aykol, S. Rühl, C. Wolverton, Npj Computational Materials 2015, 1, 15010; J. E. Saal, S. Kirklin, M. Aykol, B. Meredig, C. Wolverton, JOM 2013, 65, 1501.

[2] P. T. Anastas, J. C. Warner, *Green Chemistry: Theory and Practice*, Oxford University Press, 1998.

[3] the 25th meeting of the 12th Standing Committee of the National People's Congress, 2016.

[4] ATKearney, 2018.

[5] I. Lignos, S. Stavrakis, G. Nedelcu, L. Protesescu, A. J. deMello, M. V. Kovalenko, Nano Lett 2016, 16, 1869.

[6] R. M. Guijt, A. Dodge, G. W. K. van Dedem, N. F. de Rooij, E. Verpoorte, Lab on a Chip 2003, 3, 1.

[7] M. Abolhasani, C. W. Coley, L. Xie, O. Chen, M. G. Bawendi, K. F. Jensen, Chemistry of Materials 2015, 27, 6131.

[8] M. D. Clark, S. K. Kumar, J. S. Owen, E. M. Chan, Nano letters 2011, 11, 1976.

[9] A. P. Alivisatos, science 1996, 271, 933.

[10] C. Wagner, Zeitschrift für Elektrochemie, Berichte der Bunsengesellschaft für physikalische Chemie 1961, 65, 581.

[11] M. A. Hines, G. D. Scholes, Adv. Mater. 2003, 15, 1844.

[12] G. Frens, Nature Physical Science 1973, 241, 20.

[13] Y. Li, J. Cheng, Y. Liu, P. Liu, W. Cao, T. He, R. Chen, Z. Tang, The Journal of Physical Chemistry C 2017, 121, 5208.
